# Glucocorticoids increase adiposity by stimulating Krüppel-like factor 9 expression in macrophages

**DOI:** 10.1038/s41467-024-45477-8

**Published:** 2024-02-08

**Authors:** Yinliang Zhang, Chunyuan Du, Wei Wang, Wei Qiao, Yuhui Li, Yujie Zhang, Sufang Sheng, Xuenan Zhou, Lei Zhang, Heng Fan, Ying Yu, Yong Chen, Yunfei Liao, Shihong Chen, Yongsheng Chang

**Affiliations:** 1 Department of Physiology and Pathophysiology, School of Basic Medical Sciences, Key Laboratory of Immune Microenvironment and Disease (Ministry of Education), Tianjin Key Laboratory of Cellular Homeostasis and Disease, Tianjin Medical University, Tianjin, China; 2 Key Laboratory of Biotechnology of Hubei Province, Key Laboratory of Biotechnology of Chinese Traditional Medicine, National & Local Joint Engineering Research Center of High-throughput Drug Screening Technology, Hubei University, Wuhan, China; 3 Ningxia Key Laboratory of Stem Cell and Regenerative Medicine, General Hospital of Ningxia Medical University, Ningxia, China; 4 Department of Pharmacology, School of Basic Medical Sciences, Tianjin Medical University, Tianjin, China; 5 Department of Endocrinology, Wuhan Union Hospital, Tongji Medical College, Huazhong University of Science and Technology, Wuhan, China; 6 Department of Endocrinology, The Second Hospital of Shandong University, Jinan, China

**Keywords:** Obesity, Metabolic disorders

## Abstract

The mechanisms underlying glucocorticoid (GC)-induced obesity are poorly understood. Macrophages are the primary targets by which GCs exert pharmacological effects and perform critical functions in adipose tissue homeostasis. Here, we show that macrophages are essential for GC-induced obesity. Dexamethasone (Dex) strongly induced Krüppel-like factor 9 (*Klf9*) expression in macrophages. Similar to Dex, lentivirus-mediated *Klf9* overexpression inhibits M1 and M2a markers expression, causing macrophage deactivation. Furthermore, the myeloid-specific *Klf9* transgene promotes obesity. Conversely, myeloid-specific *Klf9*-knockout (mKlf9KO) mice are lean. Moreover, myeloid *Klf9* knockout largely blocks obesity induced by chronic GC treatment. Mechanistically, GC-inducible KLF9 recruits the SIN3A/HDAC complex to the promoter regions of *Il6*, *Ptgs2*, *Il10*, *Arg1*, and *Chil3* to inhibit their expression, subsequently reducing thermogenesis and increasing lipid accumulation by inhibiting STAT3 signaling in adipocytes. Thus, KLF9 in macrophages integrates the beneficial anti-inflammatory and adverse metabolic effects of GCs and represents a potential target for therapeutic interventions.

## Abstract (web summary)

Glucocorticoids, potent anti-inflammatory drugs, can cause or exacerbate obesity, yet the underlying molecular mechanisms remain largely unknown. Here, the authors show KLF9 in macrophages integrates the beneficial anti-inflammatory and adverse metabolic effects of glucocorticoids.

## Introduction

Dexamethasone (Dex) and other glucocorticoids (GCs) have been used clinically for decades and are the most frequently used class of anti-inflammatory agents^1^. However, chronic GC treatment has been associated with numerous adverse metabolic outcomes, including increased fat mass, central obesity, weight gain, hepatic steatosis, and hyperglycemia^2,3^.

Cortisol is the primary endogenous adrenal steroid in humans, while corticosterone (Cort) is the primary corticosteroid in rodents due to the lack of 17-α hydroxylase^4^. Two enzymes (11β-HSD1 and 11β-HSD2) are responsible for the conversion between inactive cortisone (or 11-dehydrocorticosterone in mice) and active cortisol (or Cort in mice)^4^. The action of these GCs is mediated by the mineralocorticoid receptor (MR) and glucocorticoid receptor (GR)^5^. However, the synthetic glucocorticoid Dex is highly selective for and strongly activates the GR in vivo^5^.

A number of studies have suggested that GCs are directly implicated in many aspects of adipocyte biology, including adipogenesis, lipolysis, and thermogenesis^2,3^. However, the in vivo effects of GCs are complex and recent studies have challenged the notion that the adipocyte GR mediates many of these activities. Although Dex is included in virtually all typical adipogenic cocktails and the proadipogenic effects of GCs are well accepted, GR is dispensable for adipogenesis in vitro and in vivo^6–8^. In addition, adipocyte GR deficiency promotes adipose tissue (AT) expandability under GC exposure^9^. Additionally, there is considerable evidence to support the prolipolytic effects of GCs, which promote the liberation of energy substrates during stress to meet the increased metabolic demands of the body by activating the transcription of the lipase proteins ATGL and HSL; however, chronic GC treatment induces insulin resistance leading to increased insulin secretion, which, in turn, suppresses lipolysis and therefore antagonizes the effect of GCs^2,10^. Individuals with Cushing syndrome or those with chronic exogenous corticosteroid treatment exhibit increased weight gain and visceral adiposity^2^. Moreover, long-term GC treatment also increases fat mass and adiposity in mice^11,12^. GC treatment is known to increase food intake and alter food preferences in both humans and mice^10,13^. However, a recent study suggests enhanced food intake cannot fully explain GC-induced obesity^14^. Moreover, treatment of mice with Dex, which preferentially binds to the GR, increased adiposity without influencing food intake^15^. Thus, how GCs increase adiposity and weight gain remains incompletely understood.

White adipose tissue (WAT) is essential for triglyceride storage and endocrine signaling, while brown adipose tissue (BAT) is the main site for non-shivering thermogenesis in mammals. BAT produces heat through UCP1-mediated uncoupling substrate oxidation from ATP production^16,17^. Additionally, beige adipocytes have been identified in WAT and these cells undergo a browning process in response to environmental or hormonal stimuli^16^. In beige cells, SERCA2b, one of the isoforms of SERCA2 (sarco/endoplasmic reticulum Ca^2+^-ATPase 2b) encoded by the *Atp2a2* gene, controls UCP1-independent thermogenesis^18^.

Macrophages are widely distributed immune cells. Polarized macrophages can be broadly classified into two main groups: classically activated (M1) macrophages and alternatively activated (M2) macrophages, which can be further subdivided into M2a, M2b, and M2c^19,20^. M1 macrophages develop in response to stimulation with IFN-γ and LPS and secrete significant amounts of proinflammatory cytokines, including TNF-α and IL-1β^19^. M2a cells are induced by IL-4 or IL-13 and express high levels of arginase 1 (ARG1) and CCR8 as well as CCR3 ligands^19^. M2c cells are usually regarded as deactivated macrophages and are stimulated by GCs, IL-10, and TGF-β. The common hallmark of M2c cells is the downregulation of proinflammatory cytokine expression^19,20^. Similar to GCs, elevated serum levels of IL-10 and TGF-β are positively correlated with obesity^21,22^. These findings indicate a close link between macrophage deactivation and adiposity.

Of the AT immune cells, macrophages are the most abundant and can make up 40% of all stromal vascular cells in obese AT^23^. Accumulating evidence suggests that macrophages play critical roles in regulating metabolic homeostasis^24^. Macrophages play an essential role in modulating adipogenesis^25^, lipid storage^26^, and adaptive thermogenesis^27^ in AT. Resident AT macrophages (ATMs) in lean animals are generally regarded as M2 macrophages, which express immunosuppressive factors that promote macrophage clearance of dead cells and tissue remodeling^23,25^. In contrast, obesity positively correlates with the accumulation of proinflammatory M1 macrophages in AT^23,25^. Notably, M1 and M2 activation represent extremes of a continuum in a universe of activation states, and these definitions largely derive from in vitro studies^28^. ATMs exist across a polarization spectrum from the inflammatory to anti-inflammatory phenotypes^28,29^. Macrophages are one of the main targets by which GCs exert pharmacological effects in vivo^1^. However, whether and how macrophages in AT mediate the effects of GCs on AT expansion remain unexplored.

Krüppel-like factor 9 (KLF9, also called basic transcription element binding protein 1), a member of the specificity protein/Krüppel-like family of zinc-finger domain transcription factors, plays a key role in the development^30^. Notably, KLF9 is involved in thyroid hormone-dependent effects on neurite extension and branching^31^. A conserved alpha-helical repression motif (alpha-HRM) in the amino terminus of KLF9 mediates its transcriptional repression activity by recruiting the corepressor SIN3A^32^. SIN3A acts as a master transcriptional scaffold and corepressor that mediates transcriptional silencing through associated histone deacetylases (HDAC1/HDAC2)^33^. Notably, a human genetic study (genome-wide association study) indicated that *KLF9* was associated with BMI^34^.

In the present study, we showed that GCs induce *Klf9* expression in macrophages. In vitro and in vivo experiments suggested that the *Klf9* overexpression caused macrophage deactivation and reduced the secretion of factors including IL-6, prostaglandin E2 (PGE2) and IL-10, thereby inhibiting thermogenesis and increasing lipid accumulation in adipocytes by inhibiting STAT3 signaling, which eventually led to obesity. In contrast, myeloid-specific *Klf9* knockout (mKlf9KO) mice were lean. Our data showed that KLF9 activation in macrophages was a mechanism underlying the beneficial anti-inflammatory effects and adverse metabolic outcomes mediated by GCs.

## Results

### ATMs are involved in GC-induced obesity

Macrophages, which play critical roles in regulating metabolic homeostasis, are one of the main targets through which GCs exert pharmacological effects in vivo^1,24^. Notably, long-term GC treatment of wild-type (WT) mice increased fat mass and adiposity, and adipocyte GR deficiency resulted in increased fat mass in response to GC treatment^9,12^. Thus, we hypothesized that ATMs were involved in GC-induced obesity.

**Fig. 1:**
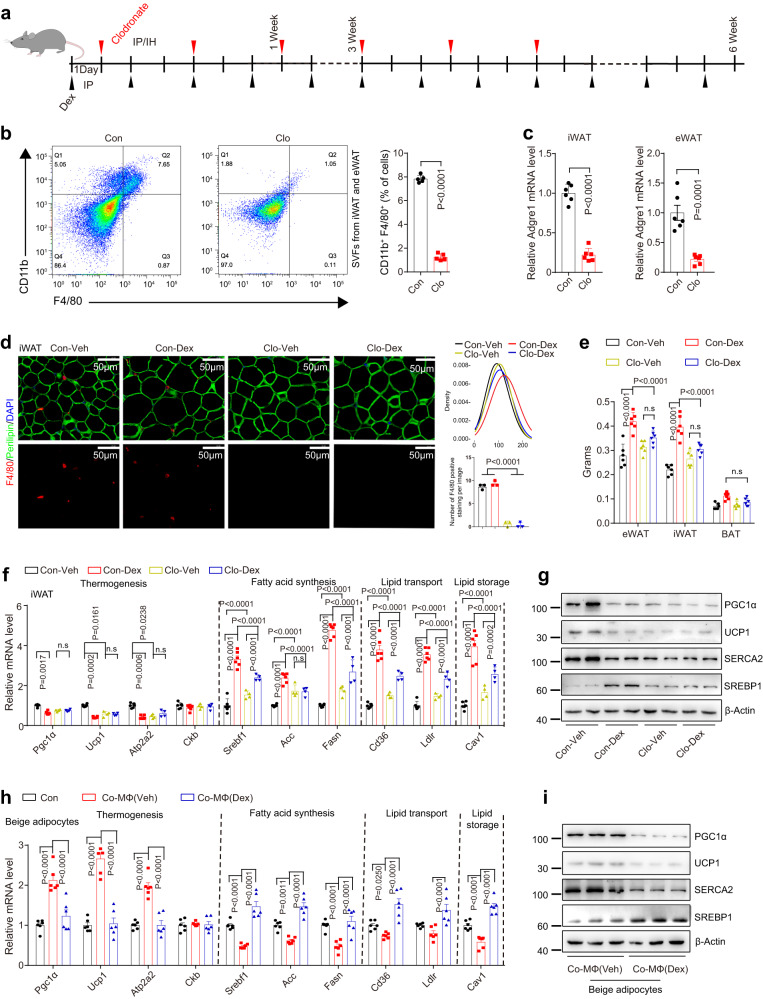
ATMs are involved in glucocorticoid-induced obesity. **a** Schematic overview showing treatment of C57BL/6J mice with dexamethasone (Dex) or clodronate-liposomes (Clo). **b** Representative histograms from flow cytometry analysis of CD11b and F4/80 expression in stromal-vascular fractions (SVFs) in adipose tissue (AT) of mice treated with Clo as in (**a**), amounts of CD11b^+^ F4/80^+^ cells are also quantified (*n* = 5 mice) scale bar = 50 μm. Control, Con. **c** *Adgre1* mRNA levels in iWAT and eWAT of mice treated as in (**b**) (*n* = 6 mice). **d** Immunofluorescence staining of Perilipin-1 in iWAT of mice treated as in (**a**), quantification of iWAT adipocyte size, and F4/80 positive staining per image are also shown (*n* = 3 mice). Vehicle, Veh. **e** AT weight of mice treated as in (**a**) (*n* = 6 mice). **f** mRNA levels of indicated genes in iWAT of mice treated as in (**a**) (Con, *n* = 6 mice; Clo, *n* = 4 mice). **g** PGC1α, UCP1, SERCA2, and SREBP1 protein levels in iWAT of mice treated as in (**a**). mRNA (**h**) (*n* = 6 independent experiments) and protein (**i**) levels of the indicated genes in differentiated mouse beige adipocytes treated with the CM from macrophages which were exposed to vehicle or Dex (100 nM) for 4 h and then co-stimulated with IL-4 (20 ng/ml) and LPS (1 ng/ml) for 12 h. Data are represented as mean ± SEM., unpaired two-tailed Student’s *t* tests were performed in (**b**) and (**c**), one-way ANOVAs were performed in (**d**), or two-way ANOVAs were performed in (**e**, **f**, and **h**).

To test this hypothesis, C57BL/6J mice were intraperitoneally injected with vehicle or Dex (every other day for 6 weeks; 5 mg/kg). Meanwhile, clodronate liposomes were administered via intraperitoneal and subcutaneous injections to deplete ATMs (3 doses during week 1 and week 3, total of 6 doses) (Fig. 1a). Flow cytometric analysis of ATMs using fluorescence-conjugated anti-CD11b and anti-F4/80 (encoded by *Adgre1* gene) antibodies indicated that clodronate liposomes administration significantly reduced the number of CD11b^+^ F4/80^+^ macrophages in subcutaneous inguinal white adipose tissue (iWAT) and epididymal white adipose tissue (eWAT) (Fig. 1b). Furthermore, quantitative PCR and immunofluorescent (IF) staining analysis confirmed that clodronate liposome treatment depleted macrophages in iWAT and eWAT (Figs. 1c, d, and S1a). Notably, we found macrophage depletion protected against chronic Dex-induced excessive AT expansion without increasing the body weight (Fig. 1e and Supplementary Fig. S1b). Consistently, IF staining revealed that long-term Dex treatment increased the size of white adipocytes in control mice but not in macrophage-depleted mice (Fig. 1d and Supplementary Fig. S1a). Mechanistically, macrophage depletion alleviated Dex-mediated induction of expression of lipid metabolism genes involved in de novo lipogenesis (*Srebf1*, *Acc*, and *Fasn*), lipid transport (*Cd36* and *Ldlr*), and lipid storage (*Cav1*), while it abolished the Dex-mediated the suppression of thermogenic genes (*Pgc1α*, *Ucp1*, and *Atp2a2*) in iWAT and eWAT (Fig. 1f and Supplementary Fig. S1c). Notably, macrophage depletion in mice also altered the protein levels of PGC1α, UCP1, SERCA2, and SREBP1 in iWAT and eWAT, mimicking the effects of Dex treatment; Moreover, Dex did not further change the protein expression of these genes in macrophage-depleted mice (Fig. 1g and Supplementary Fig. S1d). However, Dex treatment did not change the total amount of protein in iWAT and eWAT (Supplementary Fig. S1e).

Given that BAT is the main site for non-shivering thermogenesis in mice^35^, we also examined the protein level of UCP1 in BAT following Dex treatment. Although UCP1 protein per microgram protein in BAT was significantly reduced in Dex-treated mice, the total protein amount in BAT was markedly increased, leading to no change in the total amount of UCP1 protein per BAT depot (Supplementary Fig. S1f). Additionally, there was a much lower number of macrophages in BAT compared to WAT. Moreover, Dex treatment did not increase the number of macrophages in BAT (Supplementary Fig. S1g). Based on the above data, we speculated that ATMs in WAT play a crucial role in GC-induced obesity.

To determine whether macrophages directly affect adipocyte metabolism, we performed co-culture experiments (Supplementary Fig. S1h). First, we analyzed the transcriptome datasets GSE37660 from human ATMs and peripheral blood-derived monocytes (PBMCs)^36^ and GSE133127 from mouse ATMs and thioglycolate-elicited peritoneal macrophages (PMs, M0 cells)^37^. Consistent with previous reports, both human and mouse ATMs present a complex activated state, with M1 and M2a macrophage markers highly expressed, compared to their respective controls^28,29^ (Supplementary Fig. S1i). Our quantitative PCR analysis also confirmed mouse ATMs exhibited higher expression of M1 and M2a marker genes relative to mPMs (Supplementary Fig. S1j). Next, we found that mouse PMs (mPMs) treated with IL-4 and LPS for 12 h also exhibited a complex activated state, with increased expression of both M1 and M2 macrophage marker genes (Supplementary Fig. S1k). As a result, treatment of beige adipocytes differentiated from the stromal-vascular fractions (SVFs) isolated from mouse iWAT with the conditioned medium (CM) from macrophages treated with Dex in combination with IL-4 and LPS significantly increased the expression of genes involved in lipogenesis, lipid transport, and lipid storage and decreased the expression of thermogenic genes compared to the control CM (macrophages treated with IL-4 and LPS (Fig. 1h, i). Therefore, these data suggest that ATMs, at least in part, mediate the stimulatory effects of Dex on the adiposity of mice.

### KLF9 in macrophages is induced by Dex to regulate macrophage deactivation

**Fig. 2:**
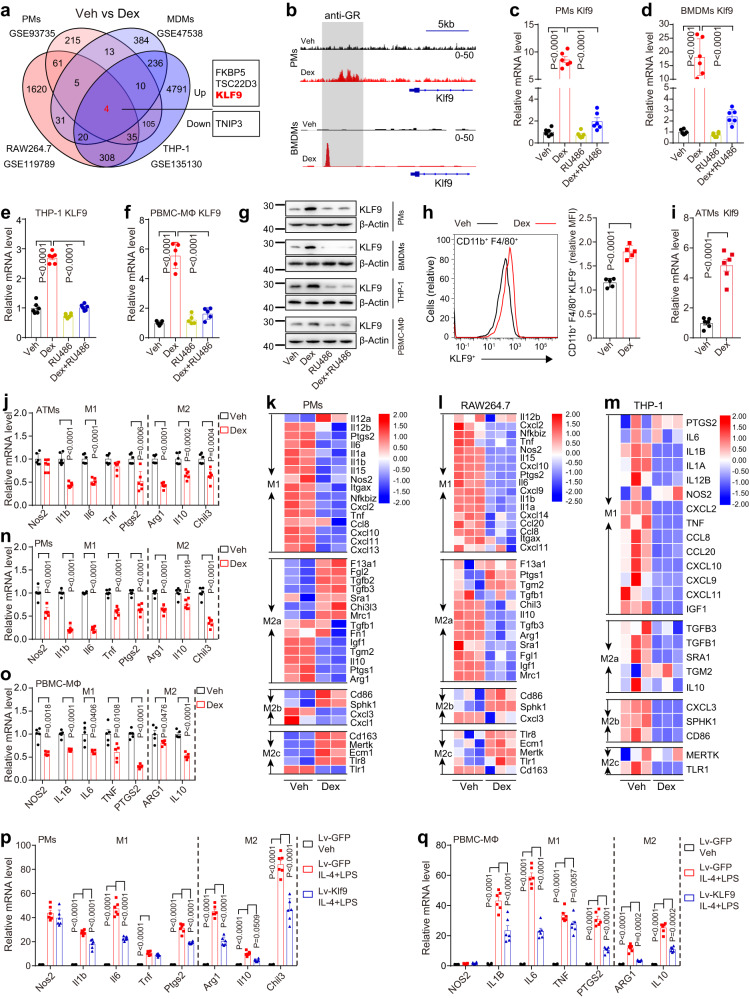
KLF9 in macrophages is induced by Dex to regulate macrophage deactivation. **a** Venn diagram showing Dex-induced differentially expressed genes identified from mPMs, RAW264.7 cells, hMDMs, and THP-1 cells under GEO number as indicated, with cutoff: |fold change|> 2, and FDR < 0.05. **b** Glucocorticoid receptor (GR) ChIP-seq on *Klf9* loci in mouse PMs and BMDMs. Genomic coordinates in mm10. *Klf9* mRNA level in mPMs (**c**) (*n* = 6 mice), mBMDMs (**d**) (*n* = 6 independent experiments), THP-1 cells (**e**) (*n* = 6 independent experiments), and hPBMC-MΦ (**f**) (*n* = 5 independent experiments) treated with Dex (100 nM) and/or GR antagonist RU486 (10 μM) for 16 h. **g** KLF9 protein level in mPMs, mBMDMs, THP-1 cells, and hPBMC-MΦ treated as in (**c**). **h** Amounts of CD11b^+^ F4/80^+^ KLF9^+^ cells in SVFs in AT of mice treated with vehicle or Dex (5 mg/kg) for 6 weeks, quantified and presented as relative mean fluorescence intensity (MFI) (*n* = 5 mice). **i** *Klf9* mRNA level in ATMs treated as in (**h**) (*n* = 6 mice). **j** mRNA levels of the indicated genes in ATMs treated as in (**h**) (*n* = 6 mice). Heat map representation of the macrophage polarization markers in mPMs (**k**), RAW264.7 cells (**l**), and THP-1 cells (**m**) treated with vehicle or Dex. mRNA levels of indicated genes in mPMs (**n**) (*n* = 6 independent experiments) and hPBMC-MΦ (**o**) (*n* = 5 independent experiments) treated with vehicle or Dex (100 nM) for 4 h and then co-stimulated with IL-4 (20 ng/ml) and LPS (1 ng/ml) for 12 h. mRNA levels of indicated genes in the mPMs (**p**) or hPBMC-MΦ cells (**q**) infected with LV-GFP or LV-Klf9 / LV-KLF9 for 24 h, then co-stimulated with IL-4 (20 ng/ml) and LPS (1 ng/ml) or vehicle for another 12 h (*n* = 6 independent experiments). Data are represented as mean ± SEM., unpaired two-tailed Student’s *t* tests were performed in (**h**, **i**, **j**, **n**, and **o**), one-way ANOVAs were performed in (**c**, **d**, **e**, and **f**), or two-way ANOVAs were performed in (**p**) and (**q**).

To examine the molecular mechanisms by which GCs regulate macrophage function, we next analyzed the publicly available transcriptome datasets GSE93735 from mPMs^38^, GSE119789 from a mouse macrophage cell line (RAW264.7)^39^, GSE47538 from human monocyte-differentiated macrophages (hMDMs)^40^ and GSE135130 from a human macrophage cell line (THP-1)^41^. The four-way Venn diagram shows 3 Dex-induced genes (*Klf9*, *Fkbp5*, and *Tsc22d3*) in mPMs, hMDMs, RAW264.7 cells, and THP-1 cells overlapped in the four datasets (Fig. 2a).

GCs exert their pharmacological effects by binding to the GR. Interestingly, Dex treatment increased GR binding to the promoter of *Klf9* but not *Tsc22d3* or *Fkbp5* in mPMs and mouse bone marrow-derived macrophages (mBMDMs) (Fig. 2b and Supplementary Fig. S2a)^42,43^. Moreover, our quantitative PCR and Western blot analysis confirmed that Dex increased the mRNA and protein levels of KLF9 in mPMs, mBMDMs, THP-1 cells, and human PBMC-derived macrophages (hPBMC-MΦ). Moreover, treatment with the GR antagonist RU486 almost completely abolished the Dex-mediated induction of KLF9 in macrophages (Fig. 2c–g). Flow cytometry revealed that the numbers of AT-infiltrated CD11b^+^ F4/80^+^ macrophages were not changed after 6 weeks of Dex treatment, but the expression of *Klf9* in ATMs was significantly upregulated (Fig. 2h and Supplementary Fig. S2b). Quantitative PCR analysis showed that chronic Dex treatment enhanced *Klf9* expression in different subsets of ATMs but not in AT (Fig. 2i and Supplementary Fig. S2cd). Thus, we hypothesize that KLF9 mediates the effects of GCs in macrophages.

Dex is well known to induce macrophage polarization toward the M2c phenotype (macrophage deactivation)^19^. As expected, chronic Dex treatment decreased the expression of the M1 and M2a macrophage markers, including *Il1b*, *Il6*, *Tnf*, *Ptgs2*, *Arg1*, *Il10*, and *Chil3*, in mouse ATMs (Fig. 2j). In addition, analysis of the GSE93735 dataset from mPMs, GSE119789 dataset from RAW264.7 cells, and GSE135130 dataset from THP-1 cells showed that Dex caused macrophage deactivation (Fig. 2k–m)^38,39^. Furthermore, quantitative PCR analysis confirmed that Dex inhibited the expression of most M1 and M2a markers, including *Nos2*, *Il1b*, *Il6*, *Tnf*, *Ptgs2*, *Arg1*, *Il10*, and *Chil3*, in mPMs with or without IL-4 and LPS stimulation (Fig. 2n and Supplementary Fig. S2e), mBMDMs (Supplementary Fig. S2f), hPBMC-MΦ (Fig. 2o), and THP-1 cells (Supplementary Fig. S2g). These data suggest that Dex induces macrophage deactivation in vivo and in vitro.

To examine the functional significance of Dex-induced *Klf9* expression in macrophages, we prepared a lentivirus expressing mouse *Klf9* (Lv-Klf9) and human *KLF9* (Lv-KLF9). Lv-Klf9 and Lv-KLF9 infection significantly increased KLF9 expression in macrophages (Supplementary Fig. S2h, i). Importantly, KLF9 overexpression in mPMs and hPBMC-MΦ strongly inhibited the expression of M1 and M2a markers with or without IL-4 and LPS stimulation, as did Dex treatment (Fig. 2p, q and Supplementary Fig. S2j, k). These results indicate that KLF9 induces macrophage deactivation.

### The myeloid-specific *Klf9* transgene promotes obesity

**Fig. 3:**
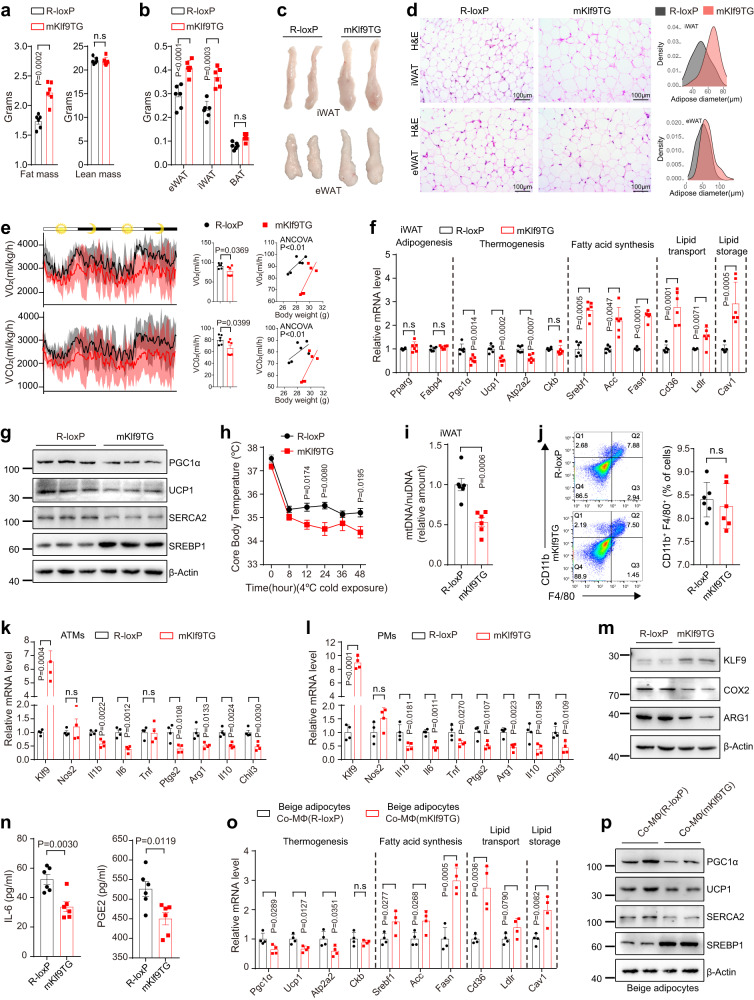
Myeloid-specific *Klf9* transgene promotes obesity. Fat and lean mass (**a**) and AT weight (**b**) of 12-week-old male mKlf9TG and R-loxP mice (*n* = 6 mice). Representative images (**c**) and H&E staining (**d**) of iWAT and eWAT from male R-loxP and mKlf9TG mice, scale bar = 100 μm, quantification of adipocyte size are also shown. Representative images of three independent experiments with similar results. **e** O_2_ consumption and CO_2_ production rates of male 12-week-old R-loxP and mKlf9TG mice normalized by body weight (left), un-normalized value of O_2_ consumption and CO_2_ production rates (middle), regression-based analysis of O_2_ consumption and CO_2_ production against body weight of R-loxP mice and mKlf9TG mice (right) (*n* = 6 mice). mRNA (*n* = 6 mice) (**f**) and protein (**g**) levels of indicated genes in iWAT of male R-loxP and mKlf9TG mice. **h** Core body temperature of male R-loxP (*n* = 5 mice) and mKlf9TG (*n* = 6 mice) mice during a cold challenge (4 °C). **i** Mitochondrial DNA (mtDNA) copy number in iWAT of male R-loxP and mKlf9TG mice (*n* = 6 mice). **j** Flow cytometry analysis of CD11b and F4/80 expression in SVFs of AT in male R-loxP and mKlf9TG mice, amounts of CD11b^+^ F4/80^+^ cells are also quantified (*n* = 6 mice). **k** mRNA levels of indicated genes in ATMs from male R-loxP and mKlf9TG mice (*n* = 4 mice). Indicated mRNA and protein levels in PMs of male R-loxP and mKlf9TG mice co-stimulated with IL-4 (20 ng/ml) and LPS (1 ng/ml) for 12 h, as measured by quantitative PCR (**l**) (*n* = 4 mice), Western blot (**m**), and ELISA (**n**) (*n* = 6 mice). Beige adipocytes treated with CM from different genotype macrophages pretreated with IL-4 (20 ng/ml) and LPS (1 ng/ml) for 12 h, mRNA (*n* = 4 independent experiments) (**o**) and protein (**p**) levels of indicated genes. Data are represented as mean ± SEM., unpaired two-tailed Student’s *t* tests were performed in (**a**, **b**, **e** (middle), **f**, **h**, **i**, **j**, **k**, **l**, **n**, **o**), or two-sided analysis of covariance (ANCOVA) was performed in (**e**) (right).

To investigate whether GC-inducible KLF9 in macrophages promotes obesity, we generated Rosa26-*Klf9*^flox/flox^ (R-loxP) mice with an insertion of the CAG-LoxP-Stop-Loxp-Klf9 cassette into the mouse *Rosa26* locus (*Klf9* knockin mice). The resulting myeloid-specific *Klf9* transgenic (mKlf9TG) mice were created by crossing *Klf9* knockin mice with Lyz2-Cre mice; littermates lacking the Cre gene (R-loxP mice) and the Lyz2-Cre mice were used as controls (Supplementary Fig. S3a–d). We found that mKlf9TG mice gained more body weight without changes in food intake and locomotor activity during normal chow diet (NCD) feeding than the control mice (Supplementary Fig. S4a and S4b). Notably, the fat pads of mKlf9TG mice were larger and weighed more than those of control mice, but there were no changes in lean mass and adipocyte numbers (Figs. 3a–c and Supplementary Fig. S4c–e). Consistently, hematoxylin and eosin (H&E) staining showed that WAT adipocytes in mKlf9TG mice were noticeably larger than those in control mice (Fig. 3d and Supplementary Fig. S4f). Thus, the increased body mass in mKlf9TG mice was attributable to increased AT weight. Biochemical analysis revealed that the serum alanine aminotransferase, aspartate aminotransferase, glucose, triglyceride, total cholesterol, and free fatty acid (FFA) concentrations were not significantly different between NCD-fed mKlf9TG mice and their controls (Supplementary Fig. S4g). In addition, NCD-fed mKlf9TG mice had normal glucose tolerance (Supplementary Fig. S4h).

We next examined potential mechanisms by which the myeloid-specific *Klf9* transgene leads to obesity. Since mKlf9TG and R-loxP mice had similar food intake and locomotor activity (Supplementary Fig. S4a), we speculated that the *Klf9* transgene impaired the thermogenesis of brown and beige fat. We found that mKlf9TG mice had a significantly lower energy expenditure, as measured by respiratory oxygen (O_2_) consumption and carbon dioxide (CO_2_) production, than R-loxP mice at room temperature (Fig. 3e). Molecular mechanism studies indicate that the *Klf9* transgene increased the expression of genes involved in lipogenesis, lipid transport, and lipid storage in iWAT and eWAT of mKlf9TG mice, whereas it did not alter expression of adipocyte differentiation marker genes, including PPARγ and Fabp4 (Fig. 3f and Supplementary Fig. S4i). Western blot analysis confirmed the increased protein levels of SREBP1, a master gene that regulates lipogenesis, in the iWAT and eWAT of mKlf9TG mice (Fig. 3g and Supplementary Fig. S4j). Of note, *Klf9* transgene did not markedly affect the size of brown adipocytes and expression of thermogenic genes in BAT (Supplementary Fig. S4k–m), possibly due to the scarcity of ATMs in BAT. In addition, myeloid *Klf9* transgene did not change the total amount of protein in BAT, iWAT, and eWAT (Supplementary Fig. S4n). Thus, this difference in energy expenditure between R-loxP and mKlf9TG mice likely reflected changes in the thermogenic activity of beige fat rather than brown fat (Fig. 3f, g and Supplementary Fig. S4i, S4j). Moreover, mKlf9TG mice failed to maintain their core body temperature when subjected to chronic cold exposure and had decreased *Ucp1* and *Atp2a2* levels in iWAT and eWAT (Fig. 3h and Supplementary Fig. S4o). Consistent with the decrease in thermogenic capacity, we found that mKlf9TG mice had fewer mitochondria in iWAT and eWAT (Fig. 3i and Supplementary Fig. S4p). Taken together, these results demonstrate that the myeloid *Klf9* transgene promotes adiposity by decreasing beige fat thermogenesis and increasing lipid accumulation in AT.

We next explored exactly how myeloid *Klf9* overexpression promoted adiposity. We found no change in CD11b^+^ F4/80^+^ cell numbers in AT between different genotypes (Fig. 3j). Thus, we speculated that KLF9 directly affected the function of macrophages, thereby promoting obesity. Next, we performed an RNA-seq analysis of mPMs from mKlf9TG and Lyz2-Cre mice. Preliminary analysis of the RNA-seq data indicated that *Klf9* transgene caused macrophage deactivation, as it inhibited the expression of some M1 and M2a macrophage-associated markers (*Il6*, *Ptgs2*, *Il10*, and *Arg1*) (Supplementary Fig. S4q). Similarly, ATMs, PMs, and BMDMs from mKlf9TG mice also exhibited a deactivated state, as measured by quantitative PCR, Western blot, and ELISA (Fig. 3k–n and Supplementary Fig. S4r, S4s).

We next examined whether macrophages with *Klf9* transgene directly affect the function of beige adipocytes. As a result, treatment of mouse beige adipocytes differentiated from SVF fraction isolated for iWAT with CM from *Klf9* transgenic macrophages increased the expression of genes involved in lipogenesis, lipid transport, and lipid storage and decreased the expression of thermogenic genes compared to the control CM (Fig. 3o, p). Because IL-6, COX2/PGE2, and M2a macrophage activation have been proven to protect against obesity^27,44,45^, our results indicate that the *Klf9* transgene in macrophages promotes obesity by inhibiting M2a macrophage polarization and IL-6 and PGE2 secretion.

### Myeloid-specific *Klf9*-deficient mice are lean

**Fig. 4:**
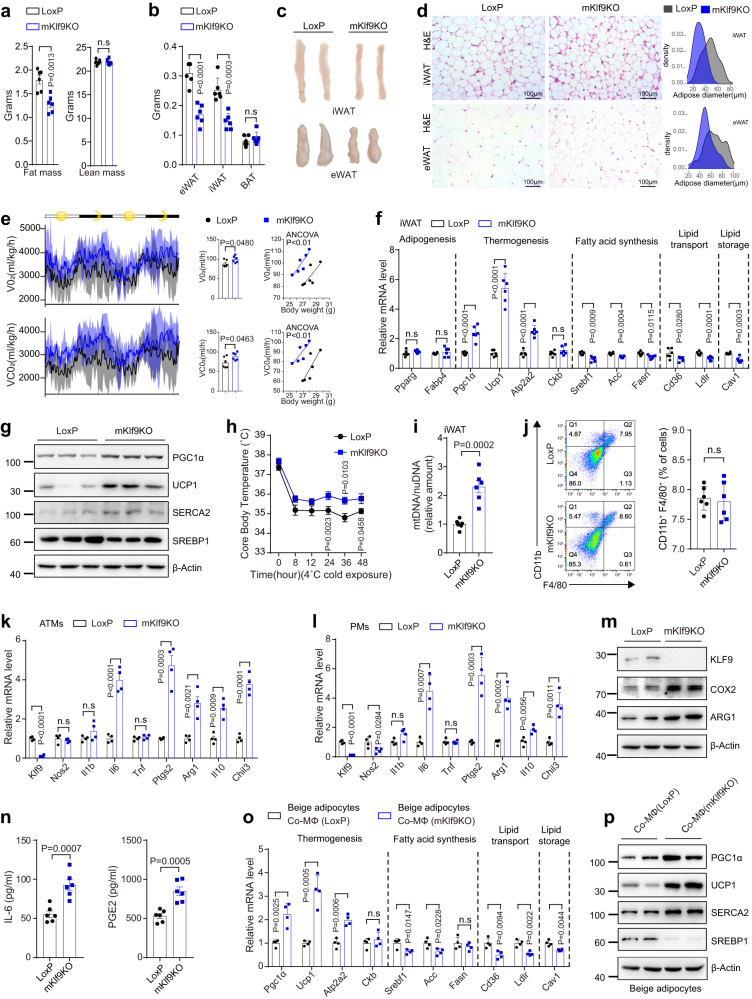
Myeloid-specific *Klf9*-deficient mice are lean. Fat and lean mass (**a**) and AT weight (**b**) of 12-week-old male mKlf9KO and LoxP mice (*n* = 6 mice). Representative images (**c**) and H&E staining (**d**) of iWAT and eWAT from male LoxP and mKlf9KO mice, scale bar = 100 μm, quantification of adipocyte size of iWAT and eWAT are also shown. Representative images of three independent experiments with similar results. **e** O_2_ consumption and CO_2_ production rates of 12-week-old male LoxP and mKlf9KO mice normalized by body weight (left), un-normalized value of O_2_ consumption and CO_2_ production rates (middle), regression-based analysis of O_2_ consumption and CO_2_ production against body weight of LoxP mice and mKlf9KO mice (right) (*n* = 6 mice). mRNA (*n* = 6 mice) (**f**) and protein (**g**) levels of indicated genes in iWAT of male LoxP and mKlf9KO mice. **h** Core body temperature of male LoxP and mKlf9KO mice during a cold challenge (4 °C) (*n* = 6 mice). **i** mtDNA copy number in iWAT of male LoxP and mKlf9KO mice (*n* = 6 mice). **j** Flow cytometry analysis of CD11b and F4/80 positive cells in SVFs in AT of male LoxP and mKlf9KO mice, amounts of CD11b^+^ F4/80^+^ cells are also quantified (*n* = 6 mice). **k** mRNA levels of indicated genes in ATMs of male LoxP and mKlf9KO mice (*n* = 4 mice). Indicated mRNA and protein levels in PMs of male LoxP and mKlf9KO mice co-stimulated with IL-4 (20 ng/ml) and LPS (1 ng/ml) for 12 h, as measured by quantitative PCR (**l**) (*n* = 4 mice), Western blot (**m**), and ELISA (**n**) (*n* = 6 mice). Beige adipocytes treated with CM from different genotype macrophages pretreated with IL-4 (20 ng/ml) and LPS (1 ng/ml) for 12 h, mRNA (*n* = 4 independent experiments) (**o**) and protein (**p**) levels of indicated genes. Data are represented as mean ± SEM., unpaired two-tailed Student’s *t* tests were performed in (**a**, **b**, **e** (middle), **f**, **h**, **i**, **j**, **k**, **l**, **n**, **o**), or two-sided analysis of covariance (ANCOVA) was performed in (**e**) (right).

To further study the physiological function of KLF9 in macrophages, we next generated mKlf9KO mice by crossing *Klf9*^flox/flox^ mice (LoxP mice)^46^ with Lyz2-Cre mice; littermates lacking the Cre gene (LoxP mice) or Lyz2-Cre mice were used as controls (Supplementary Fig. S3e–h). Body weight and fat mass were decreased in mKlf9KO mice without alterations in lean mass and adipocyte numbers (Fig. 4a, b and Supplementary Fig. S5a–e). In addition, the individual fat pads of mKlf9KO mice were visibly smaller than those of control mice (Fig. 4c). Consistently, H&E staining revealed that adipocytes from mKlf9KO mice were smaller than those from control mice (Fig. 4d and Supplementary Fig. S5f).

To evaluate the potential mechanisms of body weight loss in mKlf9KO mice, we analyzed multiple physiological parameters, including appetite, energy expenditure, glucose homeostasis, lipid metabolism, and blood biochemical parameters. We found that the food intake, locomotor activity, blood biochemical parameters, and glucose tolerance in mKlf9KO mice did not differ from those of the controls under normal dietary conditions (Supplementary Fig. S5a, g, h). However, mKlf9KO mice had higher levels of energy expenditure than LoxP mice at room temperature (Fig. 4e). In addition, quantitative PCR and/or Western blot analysis indicated that myeloid *Klf9* abrogation increased the expression of thermogenic genes in iWAT and eWAT of mice under room temperature, while it did not affect the expression of adipocyte differentiation marker genes (Fig. 4f, g and Supplementary Fig. S5i, j). Furthermore, mKlf9KO mice exhibited decreased expression of genes involved in lipogenesis, lipid transport, and lipid storage in iWAT and eWAT (Fig. 4f,g and Supplementary Fig. S5i and S5j). However, *Klf9* deficiency in macrophages did not markedly change the expression of thermogenic genes and brown adipocyte morphology (Supplementary Fig. S5k–m). In addition, myeloid *Klf9* deficiency did not change the total amount of protein in BAT, iWAT, and eWAT (Supplementary Fig. S5n). Consistently, we observed a higher core body temperature and *Ucp1* and *Atp2a2* levels in iWAT and eWAT in mKlf9KO mice during chronic cold exposure (Fig. 4h and Supplementary Fig. S5o). In addition, mitochondrial numbers in the iWAT and eWAT of mKlf9KO mice increased (Fig. 4i and Supplementary Fig. S5p). Thus, macrophage-specific *Klf9* ablation mainly increased the thermogenesis of beige fat rather than brown fat. Therefore, these data suggest that myeloid-specific *Klf9* ablation leads to mouse leanness by increasing energy expenditure and decreasing lipid accumulation.

To further examine exactly how myeloid *Klf9* ablation causes mouse leanness, we calculated the number of CD11b^+^ F4/80^+^ cells in AT by flow cytometry and found no significant differences between mKlf9KO and control mice (Fig. 4j). Next, we performed an RNA-seq analysis of PMs from control and mKlf9KO mice. Interestingly, we found that macrophage *Klf9* abrogation stimulated M2a macrophage activation (*Il10*, *Arg1*, and *Chil3*) and increased the expression of *Il6* and *Ptgs2* (Supplementary Fig. S5q). Likewise, ATMs, PMs, and BMDMs from mKlf9KO mice all exhibited an activated state, with increased expression of *Il6*, *Ptgs2*, *Il10*, *Arg1*, and *Chil3* (Fig. 4k–n and Supplementary Fig. S5r, s). Notably, mouse beige adipocytes had decreased expression of genes involved in lipogenesis, lipid transport, and lipid storage and increased expression of thermogenic genes when treated with CM from *Klf9*-ablated macrophages compared to control CM (Fig. 4o, p). Moreover, mouse beige adipocytes treated with CM from IL-4-stimulated macrophages containing IL-6 (0.2 ng/ml) and PGE2 (1 μM) exhibited increased expression of thermogenesis genes (*Pgc1α*, *Ucp1*, and *Atp2a2*) and decreased expression of lipid accumulation-related genes (*Srebf1*, *Fasn*, and *Cav1*) (Supplementary Fig. S5t). Therefore, our results show that myeloid *Klf9* abrogation stimulates M2a macrophage activation and increases the production of IL-6 and PGE2 in macrophages, thereby leading to mouse leanness.

### Macrophage KLF9 regulates white adipose tissue energy homeostasis

The Lyz2-Cre line is reported to induce target gene recombination in both macrophages and neutrophils^27^. To further illustrate whether KLF9 in other types of immune cells participates in maintaining the homeostasis of AT metabolism, we performed the unilateral ATMs depletion experiments in iWAT of mice (Supplementary Fig. S6a). First, we found that clodronate treatment significantly decreased the expression of *Adgre1* (macrophages) and did not affect the expression of *Ly6g* (neutrophils), *Siglecf* (eosinophils), *Tbet* (ILC1), *Gata3* (ILC2), and *Rorc* (ILC3) expression in iWAT of myeloid-specific *Klf9* transgenic and knockout mice (i.e., clodronate-mediated macrophage depletion does not affect the number of other types of immune cells in iWAT) (Supplementary Fig. S6b). Our above data indicate that *Klf9* transgene causes macrophage deactivation. Thus, clodronate-mediated macrophage depletion had no effect of *Klf9* transgene on AT biology in mKlf9TG mice (Supplementary Fig. S6c, d, g). However, macrophage depletion reversed the *Klf9* deficiency-mediated increase in the expression of thermogenic genes and the decrease in the expression of lipid accumulation-related genes in AT of mKlf9KO mice (Supplementary Fig. S6e, f, h). In addition, analysis of the GSE93735^38^ and GSE112922^47^ datasets showed that the target genes of KLF9 in macrophages were barely expressed in neutrophils and eosinophils (Supplementary Fig. S6i, j).

Furthermore, almost all innate immune cell types exist in AT. Neutrophil-secreted elastase (encoded by *Elane*), eosinophil-secreted IL4, ILC1-secreted IFN-γ, ILC2-secreted IL5, IL13 and Met-Enk, and ILC3-secreted IL17 and IL22 have been shown to regulate AT energy homeostasis^48–50^. To ask whether macrophage KLF9 indirectly regulates AT metabolism by affecting the function of neutrophils, eosinophils, or ILCs in vivo, we evaluated the expression of *Elane*, *Infg*, *Il5*, *Il13*, *Met-Enk*, *Il17*, and *Il22* in iWAT. However, myeloid *Klf9* transgene or knockout did not significantly alter the expression of these genes (Supplementary Fig. S6k, l). These results show that neutrophils, eosinophils, or ILCs are unlikely to be involved in the macrophage KLF9-dependent regulation of AT energy homeostasis.

Recent studies have shown that the Lyz2-Cre mouse line may result in the germline recombination of floxed alleles in a subset of neurons in the motor cortex and cerebellum^51^. Additionally, it is well known that the sympathetic nerve controls brown and beige fat thermogenesis. We, therefore, evaluated mRNA and protein levels of KLF9 in the brains of mKlf9TG, mKlf9KO, and their corresponding control mice (Supplementary Fig. S3c, d, g, h). These results showed that Lyz2-Cre mediated recombination did not significantly alter KLF9 expression in the brain and hypothalamus of mKlf9TG and mKlf9KO mice. Furthermore, IF staining for tyrosine hydroxylase (TH) suggested that myeloid-specific *Klf9* transgene or knockout did not influence sympathetic parenchymal nerve fibers activity (Supplementary Fig. S7a). We also performed unilateral denervation of the iWAT of Lyz2-Cre and mKlf9KO mice with 6-OHDA (Supplementary Fig. S7b). We found that sympathetic denervation dramatically reduced TH staining in iWAT (Supplementary Fig. S7c). However, the denervation did not influence the macrophage *Klf9* deficiency-mediated increase in the expression of thermogenesis genes and the decrease in the expression of lipid accumulation-related genes in iWAT (Supplementary Fig. S7d, e). Thus, the increased adiposity in mKlf9KO mice is not attributed to *Klf9* deficiency in neurons.

### GC-inducible KLF9 promotes macrophage deactivation by recruiting the chromatin silencer SIN3A\HDAC complex

Our above data indicate that KLF9 controls macrophage activation. We next examined the molecular mechanisms by which KLF9 inhibits the expression of M1 and M2a marker genes.

**Fig. 5:**
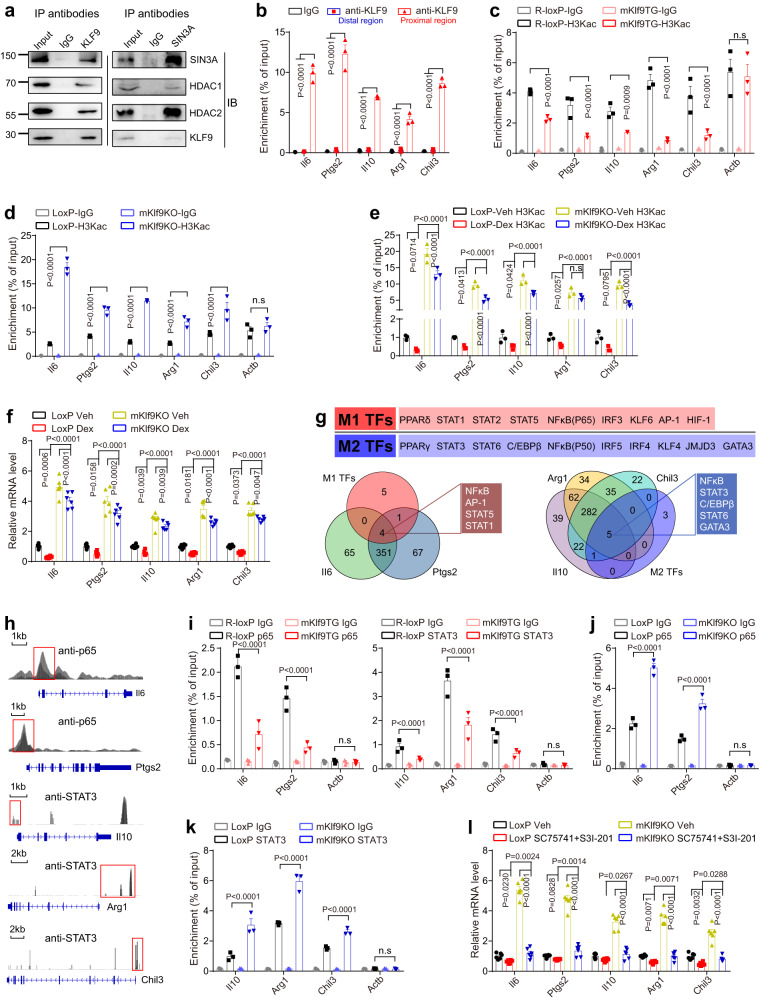
GC-inducible KLF9 promotes macrophage deactivation by recruiting the chromatin silencer SIN3A\HDAC complex. **a** Association of KLF9 with the SIN3A/HDACs complex in mPMs. Whole-cell lysates were immunoprecipitated (IP) with antibodies against the indicated proteins, then immunoblotted (IB) using antibodies against the indicated proteins. Representative images of three independent experiments with similar results. **b** The recruitment of KLF9 to the indicated gene promoters in mPMs (*n* = 3 independent experiments). Total histone H3K acetylation levels in the promoters of indicated genes in mPMs from mKlf9TG (**c**), mKlf9KO (**d**) and their corresponding control mice, co-stimulated with IL-4 (20 ng/ml) and LPS (1 ng/ml) for 12 h (*n* = 3 mice). **e** Total histone H3K acetylation levels in the promoters of indicated genes in mPMs from mKlf9KO and control mice treated with Dex (100 nM) for 4 h and then co-stimulated with IL-4 (20 ng/ml) and LPS (1 ng/ml) for 12 h (*n* = 3 mice). **f** mRNA levels of indicated genes in mPMs treated as in (**e**) (*n* = 6 mice). **g** Venn diagram showing the overlap of transcription factors predicted by Promo databases and the known transcription factors to regulate macrophage activation. **h** Genome browser shots of p65 and STAT3 ChIP-seq on indicated genes loci in macrophage. Genomic coordinates in mm10, data from GSE225833 and GSE86169. **i** The recruitment of p65 in the promoters of indicated genes in PMs from R-loxp and mKlf9TG mice (*n* = 3 mice). **j**, **k** The recruitment of STAT3 in the promoters of indicated genes in PMs from Loxp and mKlf9KO mice (*n* = 3 mice). **l** mRNA levels of indicated genes in mPMs stimulated with IL-4 (20 ng/ml) and LPS (1 ng/ml) for 12 h and treated with vehicle, p65 inhibitor (SC75741) (10 μM), or STAT3 inhibitor (S3I-201) (100 μM) for 12 h (*n* = 6 independent experiments). Data are represented as mean ± SEM., two-way ANOVAs were performed.

KLF9 contains three C2H2 zinc finger motifs in the C-terminal region and a SIN3A-binding motif in the N-terminal region^32^. Furthermore, SIN3A recruits HDACs to form the SIN3A/HDAC complex, thereby inhibiting histone acetylation levels^33^. First, we confirmed that KLF9 interacted with SIN3A/HDAC complex in mPMs, as revealed by co-immunoprecipitation (Co-IP) experiments. IP with antibodies against KLF9 followed by immunoblotting (IB) with antibodies against SIN3A, HDAC1, or HDAC2 demonstrated that KLF9 co-immunoprecipitated with the SIN3A/HDAC complex (Fig. 5a). Reciprocally, IP with antibodies against SIN3A antibodies and IB with antibodies against SIN3A, HDAC1, HDAC2, or KLF9 revealed that the SIN3A/HDAC complex co-immunoprecipitated with KLF9 (Fig. 5a). These data strongly suggest that KLF9 associates with the SIN3A/HDAC complex in mPMs. Therefore, KLF9 may act as an adapter to recruit epigenetic factors to control downstream target gene expression.

Moreover, analysis of the JASPAR database indicated that the upstream promoter regions of *Il6*, *Ptgs2*, *Il10*, *Arg1*, and *Chil3* contain binding sites for KLF9 (Supplementary Fig. S8a). As expected, chromatin immunoprecipitation (ChIP) assays confirmed that KLF9 proteins bind to the promoter regions of *Il6*, *Ptgs2*, *Il10*, *Arg1*, and *Chil3* (Fig. 5b). Furthermore, *Klf9* overexpression reduced the acetylation levels of histone H3 and H3K27 in the promoters of *Il6*, *Ptgs2*, *Il10*, *Arg1*, and *Chil3* in macrophages (Fig. 5c and Supplementary Fig. S8b). In contrast, *Klf9* deficiency in macrophages enhanced the acetylation levels of histone H3 in the promoters of these genes (Fig. 5d and Supplementary Fig. S8c). Remarkably, GEO datasets analysis indicated that GC treatment reduced the acetylation levels of histone H3K27 in the promoters of *Il6*, *Ptgs2*, *Il10*, *Arg1*, and *Chil3* in macrophages; however, no GC-response elements (GRE) binding sites were found on these gene promoters (Supplementary Fig. S8d, e)^42,52^. In addition, Dex treatment did not affect the transcription of HDACs in mPMs (Supplementary Fig. S8f, g). These findings prompted us to consider whether KLF9 is involved in GC-induced histone deacetylation. Of note, Dex significantly reduced the acetylation levels of global histone H3 and H3K27 in the promoters of *Il6*, *Ptgs2*, *Il10*, *Arg1*, and *Chil3* in control macrophages, while the extent of reduction was less in *Klf9*-deficient macrophages, as revealed by ChIP assays (Fig. 5e and Supplementary Fig. S8h). Likewise, quantitative PCR analysis showed that macrophage *Klf9* deficiency largely alleviated the inhibitory effects of Dex on the expression of *Il6*, *Ptgs2*, *Il10*, *Arg1*, and *Chil3* in mPMs (Fig. 5f).

We next explore the molecular mechanism responsible for KLF9 controlling its downstream target genes in macrophages. Using the PROMO program, we identified the NF-κB (p65), AP-1, STAT1, and STAT5 binding motif in the *Il6* and *Ptgs2* promoter region, and the NF-κB (p50), STAT3, C/EBPβ, STAT6, and GATA3 binding motif in the *Il10*, *Arg1*, and *Chil3* promoter region (Fig. 5g). A recent study suggests that p65 and STAT3 cooperate to promote macrophage activation^53^. Analysis of GSE225833^54^ and GSE86169^55^ datasets confirmed that p65 binds to the promoter region of *Il6* and *Ptgs2*, as well as STAT3 binds to the promoter region of *Il10*, *Arg1*, and *Chil3* in macrophages (Fig. 5h). Thus, we evaluated whether p65 and STAT3 are involved in KLF9-mediated macrophage deactivation. The ChIP assays showed that *Klf9* overexpression reduced p65 binding to the promoter region of *Il6* and *Ptgs2*, as well as STAT3 binding to the promoter region of *Il10*, *Arg1*, and *Chil3* in mPMs (Fig. 5i). Conversely, *Klf9* deficiency in macrophages resulted in the opposite effect (Fig. 5j, k). Notably, co-treatment with the p65 inhibitor and STAT3 inhibitor reduced the expression of *Il6*, *Ptgs2*, *Il10*, *Arg1*, and *Chil3* in control mPMs and, to a greater extent, in *Klf9* knockout mPMs (Fig. 5l). However, *Klf9* gene manipulation did not affect p65 and STAT3 phosphorylation in macrophages (Supplementary Fig. S8i, j). Based on these data, we proposed a model of KLF9 action in macrophage: GC-inducible KLF9 recruits the SIN3A/HDAC complex to the promoters of M1 and M2a marker genes, including *Il6*, *Ptgs2*, *Il10*, *Arg1*, and *Chil3*, to reduce the acetylation of H3 and H3K27, thereby inhibiting p65 and STAT3 binding to the promoter regions and expression of these target genes, subsequently leading to macrophage deactivation.

### Myeloid *Klf9* ablation alleviates the GC-induced increase in adiposity

**Fig. 6:**
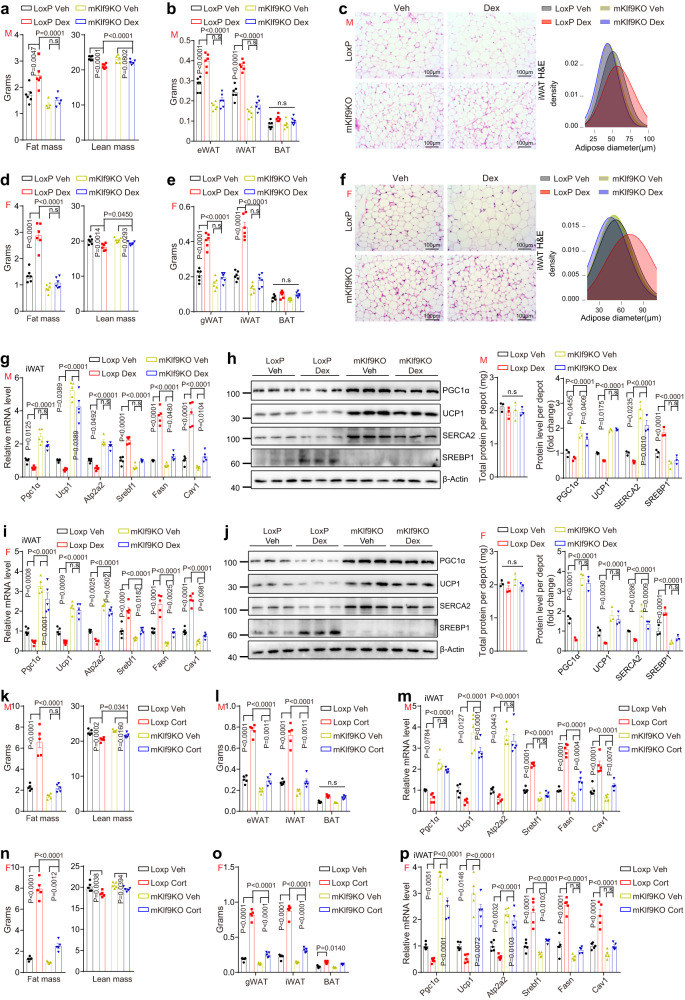
Myeloid *Klf9* ablation alleviates the GC-induced increase in adiposity. Fat and lean mass (**a**) and AT weight (**b**) of male LoxP and mKlf9KO mice treated with Dex (5 mg/kg) or vehicle for 6 weeks (Veh, *n* = 6 mice; Dex, *n* = 5 mice). (male = M). Representative images of H&E staining of iWAT of male LoxP and mKlf9KO mice treated as in (**a**) (left), scale bar = 100 μm, quantification of adipocyte size of iWAT is also shown (right). **c** Representative images of three independent experiments with similar results. Fat and lean mass (**d**) and AT weight (**e**) of female LoxP and mKlf9KO mice treated as in (**a**) (*n* = 6 mice). (female = F). **f** Representative images of H&E staining of iWAT of female LoxP and mKlf9KO mice treated as in (**a**) (left), scale bar = 100 μm, quantification of adipocyte size of iWAT is also shown (right). Representative images of three independent experiments with similar results. mRNA (*n* = 5 mice) and protein levels of indicated genes in iWAT of male (**g**, **h**) and female (**i**, **j**) LoxP and mKlf9KO mice treated as in (**a**). Fat and lean mass (**k**) and AT weight (**l**) of male LoxP and mKlf9KO mice treated with 50 μg/mL corticosterone (Cort) or corresponding vehicle (0.25% EtOH) in their drinking water for 6 weeks (*n* = 5 mice). **m** mRNA levels of indicated genes in iWAT of male LoxP and mKlf9KO mice treated as in (**k**) (*n* = 5 mice). Fat and lean mass (**n**) and AT weight (**o**) of female LoxP and mKlf9KO mice treated as in (**k**) (*n* = 5 mice). **p** mRNA levels of indicated genes in iWAT of female LoxP and mKlf9KO mice treated as in (**k**) (*n* = 5 mice). Data are represented as mean ± SEM., one-way ANOVAs were performed in (**a**, **d**, **h** (Middle), **j** (Middle), **k**, and **n**), or two-way ANOVAs were performed in (**b**, **e**, **g**, **h** (right), **i**, **j** (right), **l**, **m**, **o**, and **p**).

Long-term GC treatment of mice increases adiposity^12^. To determine whether GC-inducible KLF9 in macrophages mediates the effects of GC on adiposity, both male and female mKlf9KO mice, as well as corresponding controls, were injected intraperitoneally with Dex (5 mg/kg) or vehicle every other day for 6 weeks. First, we found that chronic Dex treatment increased the fat mass of control mice regardless of sex, while myeloid-specific *Klf9* ablation alleviated these effects of Dex (Fig. 6a, b, d, and e). Consistently, H&E staining revealed that Dex treatment increased adipocyte size in the iWAT and/or eWAT of control mice but not in mKlf9KO mice (Fig. 6c, f and Supplementary Fig. S9a). As expected, myeloid *Klf9* ablation alleviated the decrease in expression of thermogenic genes (*Pgc1α*, *Ucp1*, and *Atp2a2*) and increase in expression of lipid accumulation-related genes (*Srebf1*, *Fasn*, and *Cav1*) caused by Dex treatment in iWAT and/or eWAT (Fig. 6g, h, i, and j and Supplementary Fig. S9b). Of note, *Klf9* deficiency or Dex treatment did not affect the total amount of protein in iWAT (Fig. 6h, j). Meanwhile, myeloid *Klf9* deficiency also relieved Dex-induced hyperglycemia and hyperlipidemia (Supplementary Fig. S9c–f).

Given the difference in glucocorticoid biology between humans and rodents (mainly cortisol in humans and Cort in rodents) and synthetic glucocorticoid Dex preferentially binding to the GR over the MR in vivo, we also investigated whether macrophage KLF9 mediates Cort effect on adiposity. First, we treated male and female mKlf9KO mice as well as control mice with 50 μg/mL Cort or vehicle in their drinking water for 6 weeks. Cort treatment significantly increased fat mass and reduced lean mass in the male and female control mice, but myeloid-specific *Klf9* ablation alleviated Cort-induced adiposity (Fig. 6k, l, n, and o). Analysis of the GSE153934 transcriptome dataset^56^ showed that Cort treatment decreased the expression of thermogenic genes (*Pgc1α*, *Ucp1*, and *Atp2a2*) and increased the expression of lipid accumulation-related genes (*Srebf1*, *Fasn*, and *Cav1*) in AT, as did Dex treatment (Supplementary Fig. S9g). Our quantitative PCR results also confirmed these results (Fig. 6m, p). Importantly, myeloid-specific *Klf9* ablation mitigated the Cort-mediated multiple aspects of AT biology (Fig. 6m, p). These results indicate that myeloid KLF9 mediates the GC-induced increase in adiposity.

### GC-induced macrophage deactivation contributes to adiposity by inhibiting STAT3 signaling in adipocytes

**Fig. 7:**
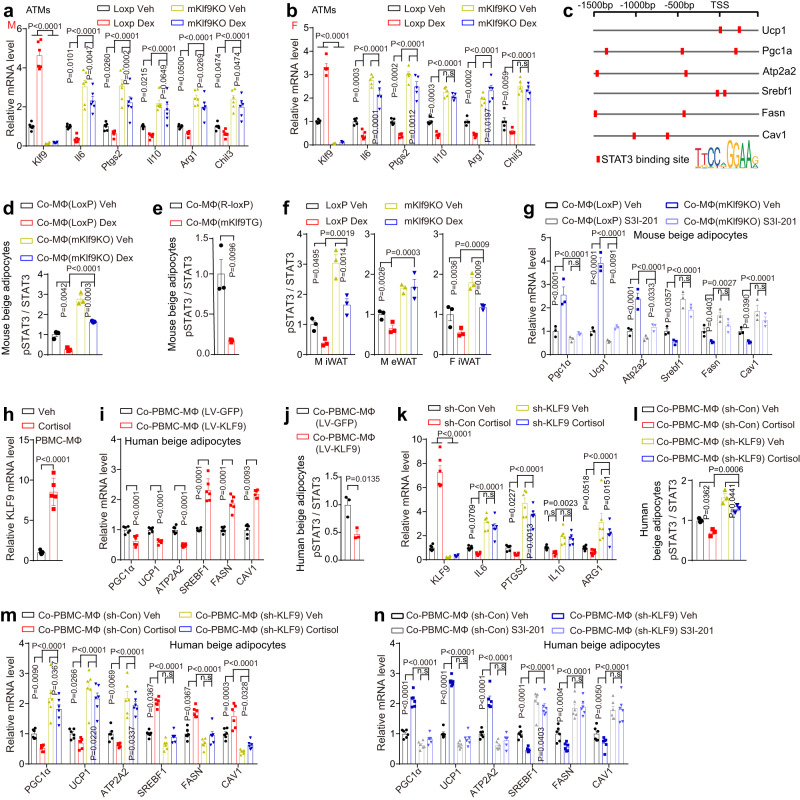
GC-induced macrophage deactivation contributes to adiposity by inhibiting STAT3 signaling in adipocytes. mRNA levels in ATMs of male (**a**), female (**b**) LoxP, and mKlf9KO mice treated with Dex (5 mg/kg) (*n* = 5 mice) or vehicle (*n* = 6 mice) for 6 weeks. **c** STAT3 binding site analysis of indicated genes. STAT3 phosphorylation in beige adipocytes treated with CM from mPMs pretreated with Dex (100 nM) or vehicle for 4 h (**d**) (without Dex (**e**)) and then stimulated with IL-4 (20 ng/ml) and LPS (1 ng/ml) for another 12 h. (*n* = 3 independent experiments). **f** STAT3 phosphorylation in WAT treated as in (**a**) (*n* = 3 mice). **g** mRNA levels in beige adipocytes treated with CM containing S3I-201 (100 μM) from indicated macrophages pretreated with IL-4 (20 ng/ml) and LPS (1 ng/ml) for 12 h (*n* = 3 independent experiments). **h** *KLF9* mRNA in hPBMC-MΦ treated with cortisol (1 μM) or vehicle for 16 h (*n* = 5 independent experiments). **i** mRNA levels in beige adipocytes treated with CM from hPBMC-MΦ pretreated with LV-KLF9 or LV-GFP for 24 h and then stimulated with IL-4 (20 ng/ml) and LPS (1 ng/ml) for another 12 h (*n* = 6 independent experiments). **j** STAT3 phosphorylation in beige adipocytes treated as in (**i**) (*n* = 3 independent experiments). **k** mRNA levels in hPBMC-MΦ pretreated with sh-KLF9 or sh-Con for 24 h and then treated with cortisol (1 μM) or vehicle for 4 h and then co-stimulated with IL-4 (20 ng/ml) and LPS (1 ng/ml) for another 12 h (*n* = 6 independent experiments). STAT3 phosphorylation (*n* = 3 independent experiments) (**l**) and mRNA levels (*n* = 6 independent experiments) (**m**) in beige adipocytes treated with CM from hPBMC-MΦ treated as in (**k**). **n** mRNA levels in beige adipocytes treated with CM containing S3I-201 from hPBMC-MΦ treated as in (**i**) (*n* = 6 independent experiments). Data are represented as mean ± SEM., unpaired two-tailed Student’s *t* tests were performed in (**e,** **h,** **i**, and **j**), one-way ANOVAs were performed in (**d**, **f**, and **l**), or two-way ANOVAs were performed in (**a**, **b**, **g**, **k**, **m**, and **n**).

To explore how myeloid *Klf9* deficiency protects against GC-induced adiposity, we isolated ATMs from mKlf9KO and control mice. Quantitative PCR analysis showed that macrophage *Klf9* deficiency alleviated the inhibitory effect of GC on the expression of *Il6*, *Ptgs2*, *Il10*, *Arg1*, and *Chil3* in ATMs from male and female mice (Fig. 7a, b and Supplementary Fig. S10a, b). Previous studies have shown that IL-6, PGE2, and IL-10 can act through STAT3 signaling^57–59^, and the activation of STAT3 signaling can directly promote thermogenesis and inhibit lipogenesis^60,61^. Notably, we found that upstream promoters of *Ucp1*, *Pgc1α*, *Atp2a2*, *Srebf1*, *Fasn*, and *Cav1* have binding sites for STAT3 (Fig. 7c).

Moreover, treatment of mouse beige adipocytes differentiated from SVFs isolated from mouse iWAT with CM from Dex-treated WT macrophages significantly decreased STAT3 phosphorylation compared to the control CM; however, *Klf9* deficiency in macrophages reduced the inhibitory effects of Dex on STAT3 phosphorylation (Fig. 7d and Supplementary Fig. S10c). Meanwhile, the phosphorylation level of STAT3 in mouse beige adipocytes decreased when the cells were treated with CM from the macrophages of mKlf9TG mice compared to the control CM (Fig. 7e and Supplementary Fig. S10d).

We next examined the effects of KLF9 in macrophages on STAT3 signaling in WAT. Notably, chronic Dex treatment decreased STAT3 phosphorylation in iWAT and/or eWAT of male and female mice, while myeloid *Klf9* deficiency alleviated the inhibitory effects of Dex on STAT3 signaling (Fig. 7f and Supplementary Fig. S10e). Furthermore, treatment of mouse beige adipocytes with CM from *Klf9*-deficient macrophages increased the expression of thermogenic genes (*Pgc1α*, *Ucp1*, and *Atp2a2*) and decreased the expression of lipid accumulation-related genes (*Srebf1*, *Fasn*, and *Cav1*) compared to CM from WT macrophages (Fig. 7g). However, a STAT3 inhibitor blocked the effects of *Klf9* deficiency on these metabolism-related genes (Fig. 7g).

To determine whether KLF9 in human macrophages controls human beige cell metabolism, we performed the following experiments. First, we observed that cortisol treatment induced KLF9 expression in hPBMC-MΦ (Fig. 7h). Next, we purchased human adipose-derived stem cells (ADSCs), which have the capacity to differentiate into beige adipocytes. Treatment of human beige adipocytes with CM from hPBMC-MΦ infected with LV-KLF9 decreased the expression of thermogenic genes (*Pgc1α*, *Ucp1*, and *Atp2a2*) and increased the expression of lipid accumulation-related genes (*Srebf1*, *Fasn*, and *Cav1*) compared to the control CM (Fig. 7i). Meanwhile, the phosphorylation level of STAT3 in human beige adipocytes also decreased when the cells were treated with a CM from KLF9-overexpressed hPBMC-MΦ compared to the control CM (Fig. 7j and Supplementary Fig. S10f–h).

Furthermore, we generated a lentivirus encoding an shRNA specific to *KLF9* (sh-KLF9). As a result, sh-KLF9-mediated *KLF9* knockdown alleviated the inhibitory effects of cortisol on macrophage marker genes (Fig. 7k and Supplementary Fig. S10i, j). Likewise, treatment of human beige adipocytes with CM from cortisol-treated hPBMC-MΦ significantly decreased STAT3 phosphorylation; however, *KLF9* knockdown in hPBMC-MΦ reduced the inhibitory effects of cortisol on STAT3 phosphorylation (Fig. 7l and Supplementary Fig. S10k). In addition, treatment of human beige adipocytes with the CM from hPBMC-MΦ with *KLF9* knockdown increased the expression of thermogenic genes and decreased the expression of lipid accumulation-related genes compared to the control CM (Fig. 7m). However, the STAT3 inhibitor blocked the effects of *KLF9* knockdown on these metabolism-related genes (Fig. 7n). In summary, we obtained similar results in human macrophage and beige cells.

**Fig. 8:**
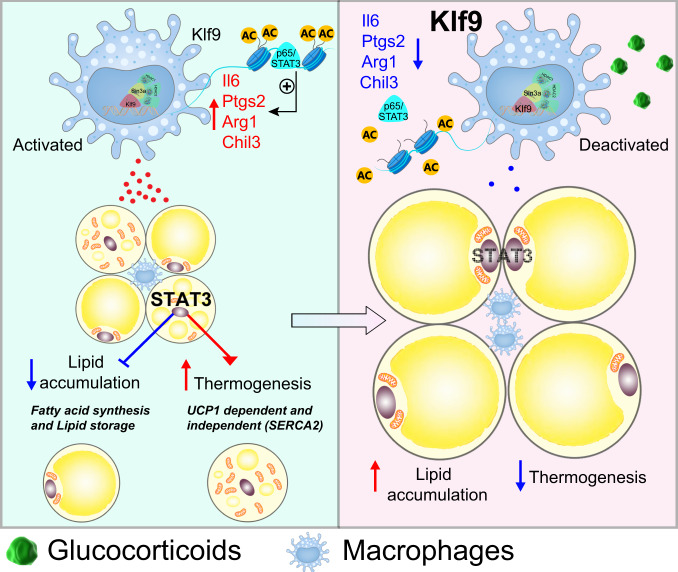
The proposed model of GC-induced adiposity. The GC/GR complex activates *Klf9* gene transcription in macrophages. KLF9 caused macrophage deactivation through recruiting chromatin silencer SIN3A/HDACs complex to the promoter region of *Il6*, *Ptgs2*, *Arg1*, and *Chil3*, thereby inhibiting p65 and STAT3 binding to the promoter regions and expression of these genes. Deactivation of macrophages leads to reduced secretion of IL6, PGE2, etc., causing decreased phosphorylation of STAT3 in adipocytes. STAT3 signaling pathway disruption in adipocytes decreased beige adipocyte thermogenesis and increased lipid accumulation, ultimately leading to adiposity.

In brief, our findings revealed that GCs induce *KLF9* gene expression, which in turn triggers macrophage deactivation by recruiting the SIN3A/HDAC complex to the promoters of *Il6*, *Ptgs2*, *Il10*, *Arg1*, and *Chil3*, thereby inhibiting p65 and STAT3 binding to the promoter regions and expression of these genes. Moreover, GC-induced macrophage deactivation disturbs the STAT3 signaling pathway in adipocytes, subsequently leading to obesity (Fig. 8). Thus, KLF9 in macrophages may be therapeutically targeted to treat GC-induced obesity.

## Discussion

Lipophilic GCs diffuse through cell membranes to bind cytosolic GRs (GRalpha and GRbeta)^1^. In the absence of ligands, GRs localize to the cytoplasm in a multiprotein complex that contains heat-shock proteins and other chaperones^1^. Ligand binding induces a conformational change in the GR that disassociates it from the chaperone complex, allowing nuclear translocation to exert biological effects^1^. Dex and other GCs are the most important and frequently used class of anti-inflammatory drugs. However, long-term GC therapy is associated with metabolic side effects, including central obesity, increased fat mass, and hyperglycemia^2,3^. Although it is well known that GCs have stimulatory effects on food intake in both humans and mice^10,13^, a recent study clearly indicates that enhanced food intake cannot fully explain GC-induced obesity^14^. Moreover, treatment of mice with Dex also enhanced adiposity without increasing food intake^15^. Additionally, adipocyte-specific GR-deficient mice gain more fat mass under GC exposure, indicating GCs stimulate adiposity in mice independently of its direct action in adipocytes^9^. These data imply an unidentified molecular mechanism for GC-induced obesity.

Macrophages are the most abundant immune cells in AT and are involved in regulating metabolic homeostasis^24^. In the present study, we show that macrophages are essential for GC-induced obesity. Moreover, we identified the activation of KLF9 in macrophages as a mechanism underlying the anti-inflammatory effects and adverse metabolic outcomes of GCs, including obesity and weight gain. Lentivirus-mediated overexpression of *Klf9* caused macrophage deactivation, which was characterized by inhibiting the expression of *Il6*, *Ptgs2*, *Il10*, *Arg1*, and *Chil3*, similar to the effect of GC treatment. Of note, mKlf9TG mice developed obesity due to decreased UCP1-dependent and UCP1-independent thermogenesis and increased lipid accumulation in adipocytes. Conversely, mKlf9KO mice were lean due to increased adaptive thermogenesis and decreased lipid accumulation in adipocytes. Moreover, myeloid-specific *Klf9* deficiency abrogated the effects of GCs on the increase in adiposity, clearly suggesting that KLF9 in macrophages has a critical role in regulating the metabolic functions of AT in response to GC treatment. These studies suggest macrophage KLF9 mediates GC stimulatory effects on adiposity via a two-pronged mechanism: increasing lipogenesis and reducing thermogenesis (UCP1-dependent and independent) in adipocytes. However, there are limitations in our study. We measured the energy expenditure of mKlf9TG and mKlf9KO mice at room temperature, but not at thermoneutrality or under cold exposure. Additionally, we just examined energy expenditure of male transgenic and knockout mice, but not female mice. These studies are required to further confirm the physiological role of macrophage KLF9 in the regulation of thermogenesis.

GCs are generally thought to suppress BAT thermogenesis. Thus, GC-mediated reduction in BAT thermogenesis contributes to GC-induced obesity. However, a recent study indicates the Cort treatment did not affect the total amount of UCP1 protein in BAT, although it decreased UCP1 protein per microgram protein in BAT^14^, consistent with our results. Moreover, Cort treatment did not influence NE-induced O_2_ consumption and energy expenditure, clearly indicating the GCs do not alter brown fat thermogenic capacity^14^. Furthermore, mice lacking GR in BAT do not affect the development of diet-induced obesity, glucose, and lipid metabolism, or food intake, indicating that GR in BAT adipocytes plays a negligible role in systemic metabolism and BAT function^62^. Our study shows that *Klf9* transgene or knockout in macrophage did not markedly alter total BAT UCP1 amounts but affected lipogenesis and thermogenesis in iWAT and eWAT. Why do not GCs significantly affect brown fat function? On the one hand, the numbers of macrophages in BAT are much less than that in WAT. On the other hand, the basal UCP1 level is much higher in BAT relative to WAT. Thus, GC-inducible KLF9 in macrophages does not markedly control brown adipocyte thermogenesis.

Tissue macrophages are often classified based on their inflammatory state as M1 proinflammatory macrophages and M2 anti-inflammatory cells^23,25^. However, in the in vivo setting, ATMs exist across a polarization spectrum from the inflammatory to anti-inflammatory phenotypes^29^. Consistent with these studies, we also found that ATMs from normal mice and humans exhibited a complex activated state and highly expressed both M1 and M2a macrophage markers, including *Il6*, *Ptgs2*, *Il10*, and others. Macrophages are major targets of GCs that limit overwhelming and sustained inflammation. We found that Dex treatment led to macrophage deactivation, inhibiting the expression of M1 and M2a macrophage markers. Actually, GCs have been previously shown to downregulate the expression of the cytokines IL-6, IL-1β, and TNF-α in myeloid cells^1^. GCs also inhibit the synthesis of enzymes, including COX2 and iNOS^63^.

Cort has been shown to increase food intake and AT macrophage infiltration^64^. However, based on previous reports and the results of our study, it appears that Dex, which mainly activates the GR rather than the MR, has no significant effect on macrophage infiltration in AT under NCD conditions and even decreases under high-fat diet conditions^65,66^. Prior studies have shown that transgenic mice overexpressing human MR exhibit increased food intake and ATMs infiltration^67,68^. Thus, Cort likely increases food intake and AT macrophage infiltration via the MR.

IL-6 is a cytokine with many physiological actions that regulate metabolism. IL-6 has been shown to increase lipolysis, glucose and fatty acid oxidation, and energy expenditure by activating the JAK1-STAT3 pathway^57^. IL-6-deficient mice develop obesity^44^. Macrophages are a rich source of cyclooxygenase and the most prevalent prostanoid produced by macrophages is PGE2. COX2, and its metabolite PGE2 have been shown to promote beige cell thermogenesis and improve AT function^45^. M2 macrophages express *Arg1* and are associated with metabolic homeostasis in lean WAT and produce IL10, an anti-inflammatory cytokine, in response to the Th2 cytokines IL4 and IL13, contributing to the maintenance of insulin sensitivity^69^. Previous studies have shown that IL-6, PGE2, and IL-10 can act through STAT3^57–59^, and the activation of STAT3 signaling can directly promote thermogenesis and inhibit lipogenesis^60,61^. Our data indicate that *Klf9*-deficient macrophages secrete increased levels of IL-6, PGE2, and IL-10, which, in turn, activate STAT3 signaling and inhibit lipid storage in adipocytes.

We previously showed that Dex stimulates hepatic gluconeogenesis by inducting the *Klf9* gene^46^. Here, we identified that GCs also induced *Klf9* expression in macrophages. *Klf9* overexpression leads to macrophage deactivation and obesity. These studies indicate that KLF9 is the key mediator of GC effects. Chronic GCs treatment induces glucose intolerance, hyperglycemia, and insulin resistance. However, myeloid-specific *Klf9* transgene or knockout did not alter glucose tolerance. Thus, GC-inducible KLF9 in macrophages is not responsible for GCs-mediated glucose metabolism. Our previous study indicates Dex-inducible KLF9 stimulates PGC1α, a master regulator of hepatic gluconeogenesis, thereby inducing hyperglycemia and glucose intolerance^46^. Of note, we have previously mentioned that the *Klf9* transgene in adipocytes promotes beige and brown fat thermogenesis^70^. Thus, adipocyte-specific *Klf9* transgenic mice are lean, and global *Klf9*-deficient mice are obese. However, in the present study, we found that mKlf9KO mice were lean due to the enhanced expression of M1 and M2a macrophage markers. We also measured *Klf9* gene expression in WAT in response to Dex treatment, and Dex increased *Klf9* expression in different subsets of macrophages, including recently identified lipid-associated macrophages (LAMs, F4/80^+^TREM2^+^)^71^. However, Dex did not significantly alter *Klf9* in BAT or WAT. Thus, KLF9 has distinct functions in different types of cells. Tissue- or cell-type-specific *Klf9* transgenic or knockout mice are required to study its physiological function.

Notably, genetic recombination using Lyz2-Cre in mice is somewhat leaky regarding neutrophils^27^. Our results indicate that *Klf9* transgene or knockout in macrophages did not markedly affect the function of neutrophils (elastase), eosinophils (IL-4), and other innate immune cells (INF-γ, IL-5, IL17, and other cytokines). Moreover, our co-culture experiments demonstrate that *Klf9* gene manipulation in macrophages directly controls beige adipocyte metabolism in vitro. Furthermore, we also performed unilateral ATM depletion and showed that macrophage KLF9 affects the function of adipocyte biology in vivo. In addition, analysis of the GEO dataset showed that these target genes of KLF9 in macrophages were barely expressed in neutrophils and eosinophils. Thus, our results indicate that KLF9 in macrophages, rather than neutrophils in AT function, contributes to obesity. Recent studies have shown that neurons exhibit Lyz2 promoter activity in vivo^51^. However, our result showed that Lyz2-Cre mediated recombination did not significantly disrupt *Klf9* expression in neurons or influence iWAT sympathetic nerve fibers activity in mKlf9TG and mKlf9KO mice. In addition, we performed unilateral denervation of the iWAT and further confirmed the effect of myeloid KLF9 on AT biology in the absence of sympathetic input.

In summary, our findings reveal that GC-inducible KLF9 in macrophages inhibits the expression of *Il6*, *Ptgs2*, *Il10*, *Arg1*, and *Chil3*, thereby inhibiting p65 and STAT3 binding to the promoter regions and expression of these target genes, subsequently leading to macrophage deactivation, increased lipogenesis, and reduced thermogenesis in adipocytes, ultimately leading to obesity. We show that KLF9 in macrophages integrates the beneficial anti-inflammatory effects and adverse metabolic effects (including obesity and weight gain) of GCs. Targeting KLF9 in macrophages could be a strategy to treat obesity and metabolic disease. Our study provides mechanistic insights into GC-mediated increases in adiposity and weight gain.

## Methods

### Ethical approvals

Human blood was obtained from healthy donors (sex: male and female), and the study protocol was approved by the Ethics Committee of the second hospital of Shandong University (permit number: KYLL-2023LW127). Written informed consent was obtained from each healthy donor included in the study. All study protocols involving the use of animals were approved by the Institutional Animal Care and Use Committee of Tianjin Medical University (permit number: TMUaMEC2021052).

### Animals

Rosa26 *Klf9*^flox/flox^ mice were generated using the CRISPR/Cas9 system to insert the CAG-LoxP-STOP-LoxP-Klf9 cassette into the mouse *Rosa26* locus. These mice were subsequently bred with Lyz2-Cre transgenic mice to create myeloid cell-specific *Klf9* knock-in mice (mKlf9TG). Myeloid cell-specific *Klf9* abrogation mice (mKlf9KO) were generated by breeding Lyz2-Cre mice with *Klf9*^flox/flox^ mice as described in our previous works^46^. Mice were genotyped by PCR; All genotypes were generated on a pure C57BL/6 background. We used a blinded fashion to collect raw data and analyze results throughout the experiment. All mice were fed a chow diet (1010001, Jiangsu Xietong Pharmaceutical Bio-engineering, Jiangsu).

### Animals treatment

All Mice were housed and kept in 12 h light and dark photoperiods. For cold exposure experiments, mice were placed in a refrigerator (4 °C) with free access to food and water. The core body temperature was monitored using a rectal probe (ThermoWorks, ALPINE, UTAH, USA). To deplete macrophages in white adipose tissue (WAT), clodronate liposomes or PBS liposomes were purchased from clodronateliposomes.org. For each animal, 50 μl clodronate liposomes (Clodronateliposomes.org) were administered subcutaneously or intraperitoneally on each side/one side (3 doses during week 1 and week 3, total of 6 doses). All animal euthanasia by CO_2_ inhalation and all animal care procedures were performed according to the guidelines of the Animal Care Committee of Tianjin Medical University.

### Cell culture

HEK293 cells were bought from ATCC (CRL-3216). Cells were cultured with DMEM supplemented with 10% FBS. THP-1 cells, a human myelomonocytic cell line (Pricella; CL-0233), were maintained in RPMI 1640 with 10% FBS and 1% P/S. Human adipose-derived stem cells (ADSCs) (Pricella, CP-H202) were maintained in preadipocyte medium (DMEM/F12 with 10% FBS, 15 mmol/L HEPES pH 7.4, 1% P/S).

### Bodyweight, food intake, body composition, and energy expenditure measurement

Bodyweight and food intake were measured weekly. Body composition (fat and lean mass) was measured by MRI (Echomri.Combo-700). For metabolic studies, 12-week-old mice were housed alone in metabolic cages (Columbus Instruments) and a free approach to food and water. O_2_ and CO_2_ concentrations were surveyed for 48 h.

### Glucose tolerance test

After 16 h of fasting, mice were injected intraperitoneally with glucose (2 g/kg). Blood glucose levels were measured from the tail vein at indicated times using a glucometer (One Touch Ultra; LifeScan Inc.).

### Quantitative PCR

Total RNA from tissues or cells was extracted with the TRIzol reagent (Invitrogen) based on the manufacturer’s instructions. The cDNA was produced using the Applied Biosystems High-Capacity cDNA Reverse-Transcription Kit using random primers. The quantitative PCR was performed using the SYBR Green PCR Master Mix (Promega) on a Roche LightCycler® 96 System. The relative expression of mRNAs was determined after normalization to 36B4 expression in the corresponding samples. The sequences of primers are shown in Supplemental Table 1.

### Protein analysis

Tissue and cell lysates were prepared using RIPA buffer (20 mM Tris-Cl pH 7.5, 140 mM NaCl, 1 mM CaCl_2_ and MgCl_2_, 10 mM NaF, 1% NP-40, 10% glycerol, 2 mM Na-Vanadate, and 1 mM PMSF). Proteins were subjected to SDS-PAGE and were then transferred onto polyvinylidene difluoride (PVDF) membranes (Millipore). Immunoblotting was performed with the following primary antibodies, according to the manufacturer’s protocol: KLF9 (Invitrogen; Cat: 701888; Clone name: 5H16L7; Dilution: 1:1000), KLF9 (Abcam; Cat: ab227920; Dilution: 1:1000), PGC1α (Millipore; Cat: AB3242; Dilution: 1:1000), UCP1 (Abcam; Cat: ab10983; Dilution: 1:1000), SERCA2(ABclonal; Cat: A11692; Clone name: ARC0679; Dilution: 1:1000), β-Actin (ABclonal; Cat: AC026; Clone name: ARC5115-01; Dilution: 1:20,000), ARG1 (ABclonal; Cat: A4923; Clone name: ARC1164; Dilution: 1:1000), SREBP1 (ABclonal; Cat: A15586; Dilution: 1:1000), HDAC1 (ABclonal; Cat: A0238; Dilution: 1:1000), HDAC2 (ABclonal; Cat: A2084; Dilution: 1:1000), SIN3A (Proteintech; Cat: 14638-1-AP; Dilution: 1:1000), STAT3 (Santa Cruz Biotechnology; Cat: sc-8019; Dilution: 1:50), P-STAT3 (Santa Cruz Biotechnology; Cat: sc-8059; Dilution: 1:50), p65 (Cell Signaling Technology; Cat: #8242; Clone name: D14E12; Dilution: 1:1000), P-p65 (Cell Signaling Technology; Cat: #3033; Clone name: 93H1; Dilution: 1:1000).

### Isolation of ATMs

Isolation of primary SVF cells from mice fat was performed using our previously published methods with minor modifications. Both iWAT and eWAT were isolated from mice and digested with 0.1% collagenase type I (Sigma-Aldrich) in SVF buffer (1.1 mM CaCl_2_, 2.7 mM KCl, 118 mM NaCl, 0.5 mM MgCl_2_, 0.4 mM NaH_2_PO_4_, 20 mM HEPES pH 7.2, 5.5 mM glucose and 1% BSA (fatty-acid free)) at 37 °C with shaking at 200 r.p.m. for 60 min. Next, digested tissues were filtered through a 70 μM nylon mesh and centrifuged for 5 min at 500 *g*. After centrifugation, the SVF pellet was resuspended in red-blood-cell lysis buffer. To isolate ATMs, SVFs were washed with PBS and incubated with CD11b^+^ and F4/80^+^, and ATMs were sorted from the SVFs using FACS. ATMs RNA was collected from the sorted cells via RNAprep pure Micro Kit (TIANGEN).

### Isolation of peritoneal macrophages

Peritoneal macrophages (PMs) were isolated 3 days after intraperitoneal injection of 4% thioglycollate (BD Diagnostics). Resident peritoneal cells were harvested by lavage with 10 ml ice-cold PBS and resuspended in RPMI 1640 with 10% FBS and 1% penicillin–streptomycin (P/S). For purification of the macrophages, peritoneal exudates were allowed to adhere for 1–2 h, after which non-adherent cells were removed with warm PBS to achieve greater than 90% purity of macrophages. PMs were activated by co-stimulation with IL-4 (20 ng/ml) and LPS (1 ng/ml) for 12 h.

### Isolation of bone marrow-derived macrophages

Bone marrow was collected from the femur and tibia of 8–11-week-old male mice. The supernatant was collected in cold PBS and filtered through a 70 μm nylon mesh to dispose of solid debris. The filtrate was centrifuged at 450 *g* for 10 min at 4 °C, and then the pellet was resuspended in 10 ml of red-blood-cell lysis buffer for 30 s. The resuspend was centrifuged as described above, and the pellet was resuspended in 20 ml of RPMI 1640, containing 10% FBS and 1% P/S, inoculated in a 100 mm dish for 4 h at 37 °C to remove resident bone marrow macrophages by adhesion. Collected supernatants were filtered and centrifuged as described above. The pellet was resuspended in RPMI 1640 supplemented with murine recombinant M-CSF (30 ng/ml) for 7 days at 37 °C and 5% CO_2_. The medium and stimulants are changed every 2 days. Differentiated macrophages were subsequently constructed into a single-cell suspension with 5 mM EDTA and plated in RPMI 1640 supplemented with 10% FBS and 1% P/S. 8–12 h later, BMDMs were activated by co-stimulation with IL-4 (20 ng/ml) and LPS (1 ng/ml) for 12 h.

### Induction of human macrophages

PBMCs were isolated from healthy blood donors by Ficoll gradient extraction. Isolated PBMCs were then cultured in RPMI 1640 supplemented with 10% FBS and 1% P/S and T and B cell populations were depleted via adhesion properties. The remaining adherent PBMCs were differentiated into macrophages with 30 ng/mL human macrophage colony stimulating factor for 72 h. THP-1 cells were differentiated into macrophage cells overnight using PMA (150 nM). PMA was then removed and THP-1 cells were activated by co-stimulation with IL-4 (20 ng/ml) and LPS (1 ng/ml) for 12 h.

### Differentiation of primary mouse beige adipocytes

For isolation of SVFs, iWAT of 4–6 week old male mice was digested for 30 min at 37 °C by collagenase I in SVF buffer. The digested cell suspension was centrifuged, and the pelleted cells were resuspended and filtered through a 75 μm nylon mesh. Then the SVF cells were cultured in DMEM/F12 containing 10% FBS and 1% P/S at 37 °C in a humidified 5% CO_2_ incubator. When grown to 90% confluency, cells were cultured in the induction medium (10% FBS, 1% P/S, 0.5 mM isobutylmethylxanthine, 125 μM indomethacin, 5 μM dexamethasone, 5 μg/ml insulin, 1 nM T3, and 1 μM rosiglitazone). Two days after induction, cells were changed to a maintenance medium (10% FBS, 1% P/S, 5 μg/ml insulin, 1 nM T3) and cultured for 6 days, at which point *Ucp1* expression could be detected.

### Differentiation of primary human beige adipocytes

For adipocyte differentiation, human ADSCs were cultured in the induction medium (1% P/S, 0.5 mM isobutylmethylxanthine, 125 μM indomethacin, 5 μM dexamethasone, 5 μg/ml insulin, 1 nM T3, 1 μM rosiglitazone, 17 μM pantothenate and 33 μM biotin) and 2% FBS for a further 12 days. Induction medium was changed every 3 days. The cells were then transferred to maintenance medium (2% FBS, 1% P/S, 5 μg/ml insulin, 1 nM T3) and cultured for 6 days.

### Co-culture experiments

For co-culture experiments, macrophages were pretreated with GCs for 4 h or lentiviruses for 24 h, followed by co-stimulation with IL-4 (20 ng/ml) and LPS (1 ng/ml) for 12 h. Then, the old medium was respirated and replaced with fresh medium. Cell were cultured for another 12 h, then the conditioned medium was collected. Treatment of beige adipocytes with conditioned medium: maintenance medium (1:1) for 24 h. The beige adipocytes were then collected for gene expression analysis.

### Metabolites and ELISA

The concentration of serum triglyceride, cholesterol, and FFA was determined using automatic monarch equipment (Peking Union Medical College Hospital, Beijing). The concentration of triacylglycerol and cholesterol in the liver was measured using a colorimetric diagnostic kit (Applygen Technologies). The concentration of IL-6 and PGE2 in the medium was measured using the ELISA kit (Mouse PGE2 ELISA KIT, Beijing Solarbio Science & Technology Co., Ltd; Mouse IL-6 ELISA Kit, ABclonal).

### Construction of lentivirus

The Full-length mouse *Klf9* (NM_010638) and human *KLF9 (*NM_001206*)* were amplified by PCR technique and cloned into pCDH lentiviral backbone vector. For the production of the *KLF9* overexpression lentivirus (LV-Klf9, LV-KLF9), the recombined lentiviral vector, identified by double digest with Xba1 and EcoR1 and DNA sequencing. Lentivirus-based short hairpin RNA (shRNA) expression constructs into the pLKO.1 lentiviral backbone vector to knockdown human *KLF9* expression (sh-KLF9). The lentiviral vector together with psPAX2 and pMD2.G was packaged in 293T cells. The Virus present in the culture supernatant was concentrated. Constitutive LV-Klf9 and LV-KLF9 were transfected into macrophages for 36–48 h. The efficiency of lentiviral infection was detected by quantitative PCR and Western blot analysis. Empty plasmid packing lentivirus was used as a control (LV-GFP or sh-Con).

### Histology analysis

Adipose tissue was fixed in 10% neutral formalin for at least 48 h at 4 °C and then desiccated and embedded in paraffin before being cut into 5 μm sections and stained with hematoxylin and eosin (H&E).

### Chromatin immunoprecipitation (ChIP)

The mouse macrophages were treated with 1% formaldehyde at room temperature for 10 min and lysed with ChIP cell lysis buffer (10 mM Tris-HCl, pH 8.0, 10 mM NaCl, 3 mM MgCl_2_, 0.5% NP-40, protease inhibitor cocktail, and TSA (Selleck)) and ChIP nuclear lysis buffer (50 mM Tris-HCl, pH 8.0, 5 mM EDTA, 1% SDS, protease inhibitor cocktail, and TSA (Selleck)). The cell lysates were sonicated to shear the chromatin. For KLF9 immunoprecipitation, equal aliquots of tissue lysates were incubated with anti-KLF9 antibody (701888; Invitrogen) or normal rabbit IgG overnight at 4 °C. Then tissue lysates were incubated with protein A/G-conjugated agarose beads at 4 °C for 4 h. Beads were washed with RIPA buffer and PBS for three times separately. As described above, Acetyl-Histone H3 immunoprecipitation was performed using an anti-Acetyl-Histone H3 antibody (A17917; ABclonal) or anti-Acetyl-Histone H3-K27 antibody (A2771; ABclonal); p65 and STAT immunoprecipitation was performed using an anti-p65 antibody (#8242; Cell Signaling Technology) or STAT3 (#12640; Cell Signaling Technology). Quantitative PCR analysis was performed to detect the indicated locus. The sequences of primers are shown in Supplementary Table 1.

### Immunofluorescence

Adipose tissue and cells were fixed in 4% paraformaldehyde for 24 h at 4 °C before whole-mount staining. Briefly, the specimen was permeabilized with 1% Triton X-100, blocked with 1% BSA and 3% FBS in PBS, then stained with anti-F4/80 mAbs (Proteintech; Cat: 28463-1-AP; Dilution: 1:200), anti-Perilipin-1 mAbs (Cell Signaling Technology; Cat: #9349; Clone name: D1D8; Dilution: 1:200), or anti-TH (Santa Cruz Biotechnology; Cat: sc-25269; Dilution: 1:50) followed by incubation indicated with appropriate immunofluorescent secondary antibodies. Nuclei were stained with DAPI. Fluorescent signals were detected using Leica or Olympus confocal laser scanning microscope.

### Flow cytometry

The expression of cell surface markers and intracellular molecules of macrophages was determined using flow cytometry. Cells were incubated with Fc Block (BioLegend; Cat: 101302; Clone name: 93; Dilution: 1:100) and washed using PBS with 2% fetal calf serum, and then cells were stained with PE-conjugated anti-mouse CD11b (BioLegend; Cat: 101207; Clone name: M1/70; Dilution: 1:100), PE-conjugated anti-TREM2 (R&D Systems, Cat: FAB17291P; Dilution: 1:100), PE-conjugated anti-mouse CD11c (BioLegend; Cat: 117308; Clone name: N418; Dilution: 1:100), PE-conjugated anti-mouse CD206 (BioLegend; Cat: 141706; Clone name: C068C2; Dilution: 1:100), APC-conjugated anti-mouse F4/80 (BioLegend; Cat: 123116; Clone name: BM8; Dilution: 1:100), or isotype antibodies. According to the manufacturer’s instructions, the intracellular staining of KLF9 ((Biorbyt; Cat: orb9122; Dilution: 1:100)) was performed with the transcription factor staining buffer set (eBioscience). Cells were next subjected to flow cytometry analysis with a BD FACSAria IIu flow cytometer (BD Bioscience). Data were analyzed with FlowJo Software version 7.6.4.

### Statistical analysis

All data were presented as mean ±  SEM. A two-tailed, unpaired Student’s *t* test was used for pairwise comparison of genotypes or treatments. One-way ANOVA and two-ANOVA were used when comparing three or more groups, as indicated in the Figure legends and otherwise. The analysis was performed using Microsoft Excel, GraphPad Prism, and IBM SPSS statistics. *P* < 0.05 was considered to be statistically significant.

### Reporting summary

Further information on research design is available in the Nature Portfolio Reporting Summary linked to this article.

### Supplementary information

Supplementary Information

Peer Review File

Reporting Summary

### Source data

Source data

## Data Availability

The RNA-seq data used in this study are publicly available in the NCBI Gene Expression Omnibus repository under the accession numbers GSE37660, GSE133127, GSE93735, GSE119789, GSE47538, GSE135130, GSE93735, GSE112922, GSE225833, GSE86169, and GSE153934. The data used to generate the main results shown in the main figures and extended figures are available in source data. Source data are provided with this paper. All data supporting the findings of this study are available from the corresponding authors on request addressed to Dr. Yongsheng Chang, E-mail changys@tmu.edu.cn. Source data are provided with this paper.
