# Supplementary material for: Glucocorticoids increase adiposity by stimulating Krüppel-like factor 9 expression in macrophages
Source: Nat Commun. 2024 Feb 8;15:1190. doi: 10.1038/s41467-024-45477-8 (PMC10853261; doi:10.1038/s41467-024-45477-8)
Supplement: Supplementary file 1 — Supplementary Information [file 41467_2024_45477_MOESM1_ESM.pdf]

Supplementary Materials:

Glucocorticoids increase adiposity by stimulating Krüppel-like factor 9 expression in macrophages

Supplementary Figures:

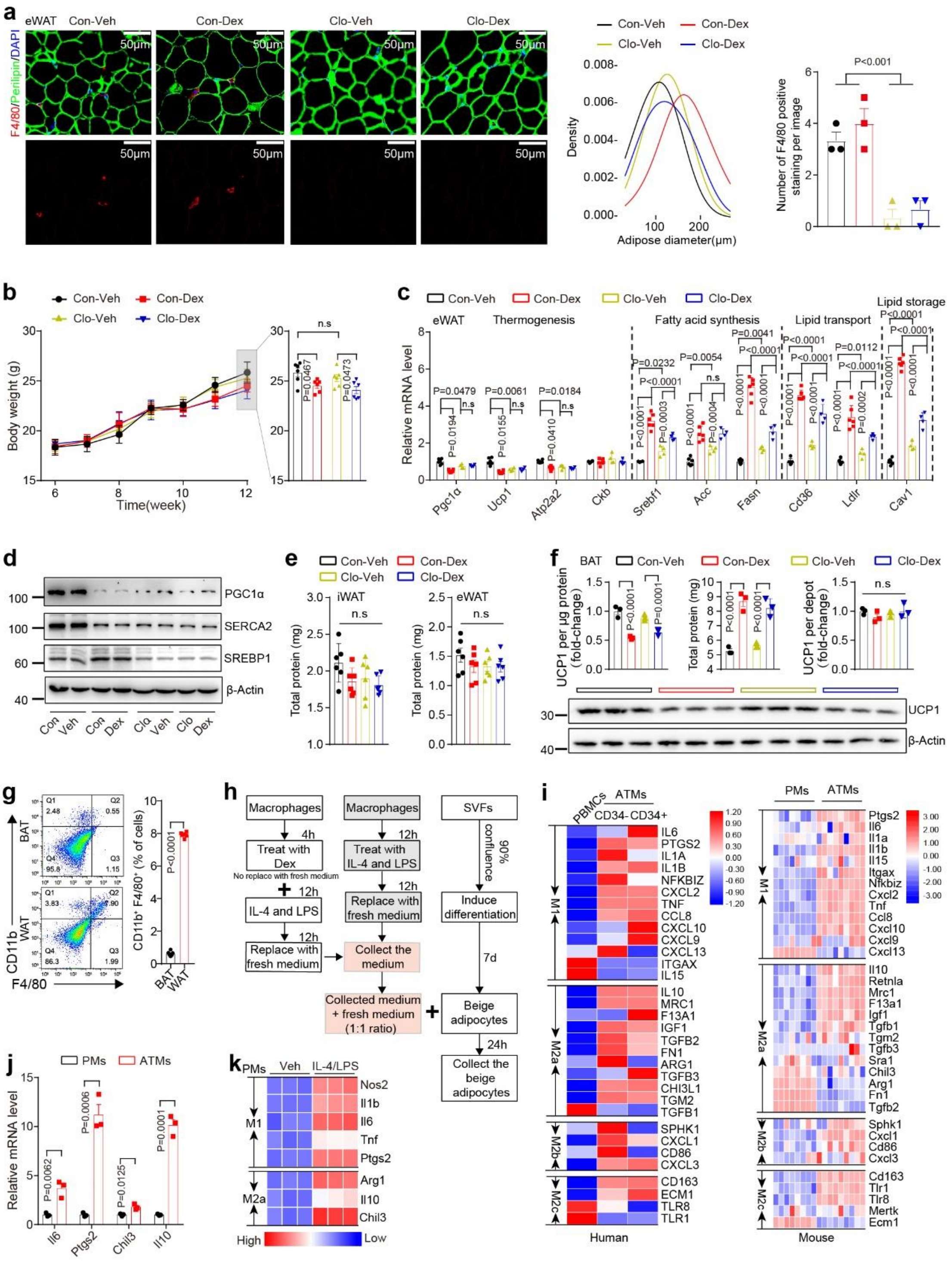

Fig. S1: ATMs are involved in GC-induced obesity.

a, Immunofluorescence staining of Perilipin-1 in eWAT of mice treated as in (**Fig. 1a**), scale bar = 50 μm, quantification of eWAT adipocyte size and quantification of F4/80 positive in per image are also shown (n = 3 mice). Representative images of three independent experiments with similar results.

b, Body weight of male mice treated as in (**Fig. 1a**) (n = 6 mice).

c, mRNA levels of indicated genes in eWAT of mice treated as in (**Fig. 1a**) (n = 6 mice).

d, protein levels of PGC1α, SERCA2, and SREBP1 in eWAT of mice treated as in (**Fig. 1a**).

e, Quantification of total protein per depot of iWAT and eWAT from mice treated as in (**Fig. 1a**) (n = 6 mice).

f, Quantification of showing UCP1 per microgram protein, total protein per depot, and total UCP1 per depot of BAT from mice treated as in (**Fig. 1a**) (n = 3 mice).

g, Representative histograms from flow cytometry analysis of CD11b and F4/80 expression in SVFs of indicated AT in male C57BL/6J mice, amounts of $CD11b^+$ $F4/80^+$ cells are also quantified (n = 6 mice).

h, Schematic depicting the treatment of SVF-derived beige adipocytes with indicated CM from macrophages.

i, Heat map representation of macrophage polarization markers in human PBMCs and ATMs (left, data from GSE37660), mPMs and ATMs (right, data from GSE133127).

j, mRNA levels of macrophage polarization markers in mPMs and ATMs (n = 3 mice).

k, mRNA levels of polarization markers of mPMs treated with vehicle or IL-4 (20 ng/ml) and LPS (1 ng/ml) for 12 h (n = 3 mice).

Data are represented as mean ± SEM., unpaired two-tailed Student's t tests were performed in **g** and **j**, one-way ANOVAs were performed in **a**, **b**, **e**, and **f**, or two-way ANOVAs were performed in **c.**

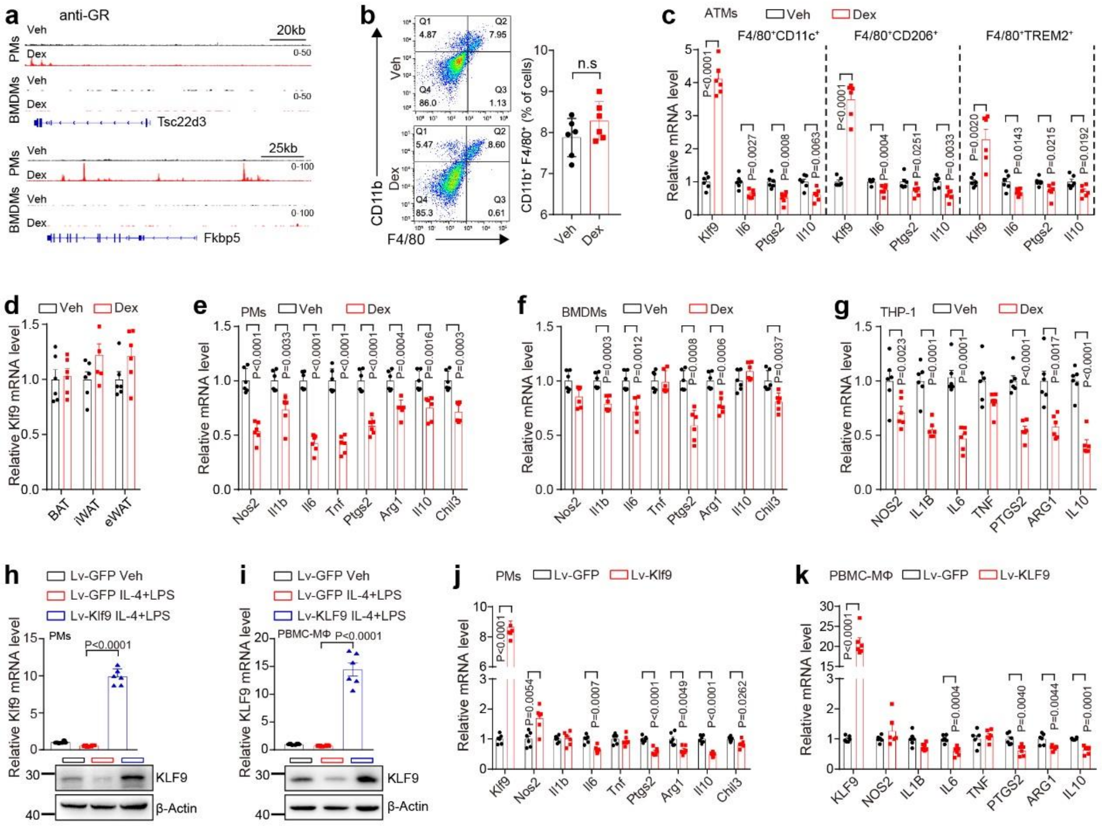

Fig. S2: Dex treatment and *Klf9* overexpression promote macrophage deactivation.

a, Genome browser shots of the GR ChIP-seq on *Tsc22d3* and *Fkbp5* loci in mPMs (GSE109131) and mBMDMs (GSE61877) treated with Dex or vehicle (Veh). Genomic coordinates in mm10.

b, Representative flow cytometry histograms from analysis of CD11b and F4/80 expression in SVFs of AT of mice after 6 weeks Dex exposure, amounts of CD11b$^+$ F4/80$^+$ cells are also quantified (n = 5 mice).

c, mRNA levels of indicated genes in different ATMs subgroups treated with vehicle or Dex (5 mg/kg) for 6 weeks (n = 6 mice).

d, *Klf9* mRNA levels of indicated AT treated with vehicle or Dex (5 mg/kg) for 6 weeks (n = 6 mice).

e-g, mRNA levels of polarization markers of mPMs (**e**). mBMDMs (**f**), and THP-1 (**g**) treated with Dex (100 nM) or vehicle for 16 h (n = 6 independent experiments).

h, **i**, KLF9 mRNA and protein levels in the mPMs (**h**) and hPBMC-MΦ (**i**) infected with LV-GFP or LV-Klf9 / LV-KLF9 for 24 h, then co-stimulated with IL-4 (20 ng/ml) and LPS (1 ng/ml) or vehicle for another 12 h (n = 6 independent experiments).

j, **k**, mRNA levels of indicated genes in the mPMs (**j**) and hPBMC-MΦ (**k**) infected with LV-GFP or LV-Klf9 / LV-KLF9 for 36 h (n = 6 independent experiments).

Data are represented as mean ± SEM., unpaired two-tailed Student's t tests were performed in **b**, **c**, **d**, **e**, **f**, **g**, **j**, and **k**, or one-way ANOVAs were performed in **h** and **i**.

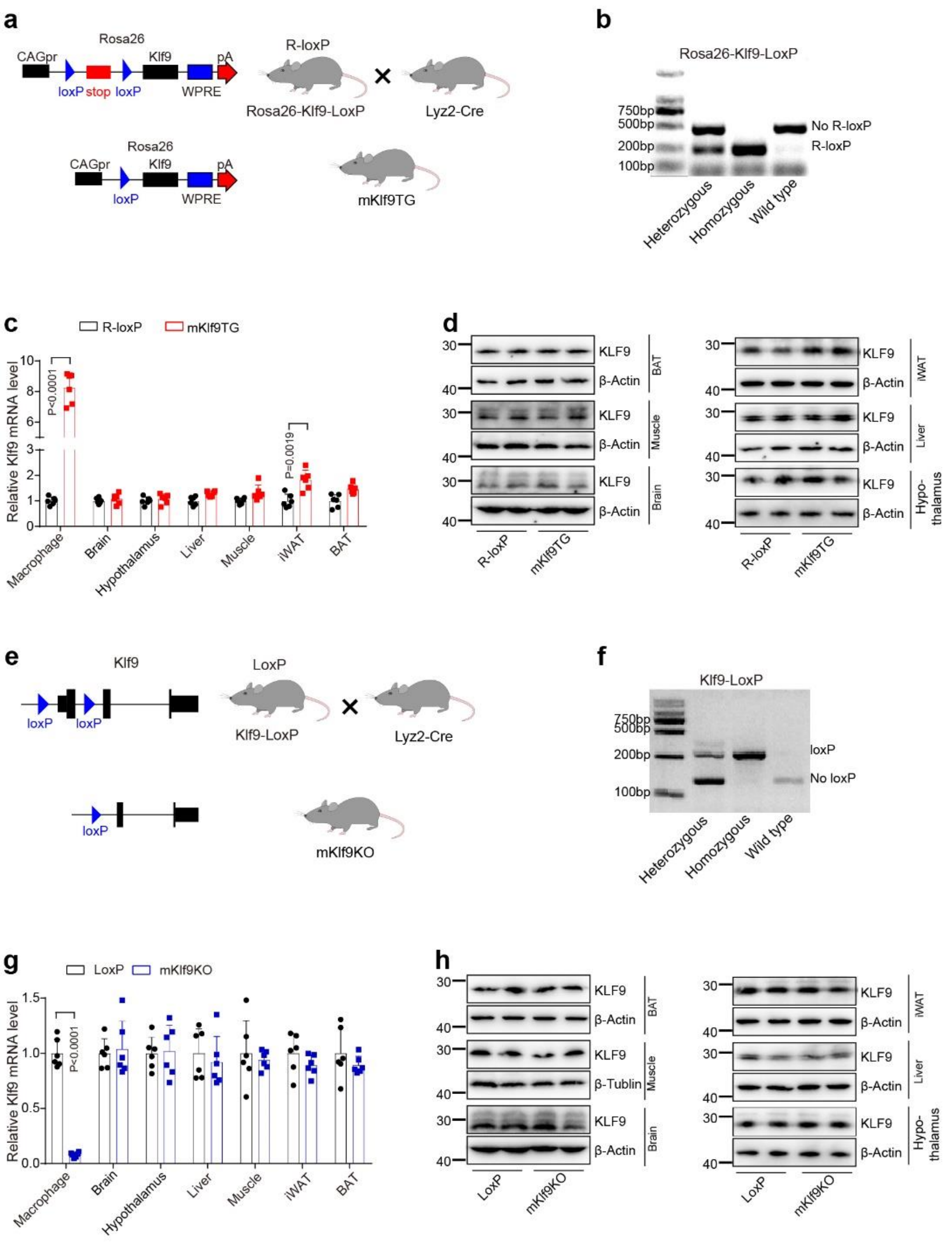

Fig. S3: Generation and characterization of macrophage-specific *Klf9* transgene and knockout mice.

a, Generation of mKlf9TG mice. Rosa26 *Klf9*$^{flox/flox}$ mice were generated using the CRISPR/Cas9 system to insert the CAG-LoxP-STOP-LoxP-Klf9 cassette into the mouse *Rosa26* locus. These mice were subsequently bred with Lyz2-Cre transgenic mice to obtain mKlf9TG mice, leading to macrophage-specific *Klf9* overexpression.

b, PCR-based genotyping of mice.

c, **d**, KLF9 mRNA (**c**) and protein (**d**) levels in the indicated tissues from male R-loxP and mKlf9TG mice (n = 6 mice).

e, Generation of mKlf9KO mice. *Klf9*$^{flox/flox}$ mice were generated using CRISPR/Cas9 technology to insert two loxP sites into exon1 of the *Klf9* gene. These mice were subsequently bred with Lyz2-Cre transgenic mice to obtain mKlf9KO mice, leading to macrophage-specific *Klf9* abrogation.

f, PCR-based genotyping of mice.

g, **h**, KLF9 mRNA (**g**) and protein (**h**) levels in the indicated tissues from male LoxP and mKlf9KO mice (n = 6 mice).

Data are represented as mean ± SEM., unpaired two-tailed Student's t tests were performed**.**

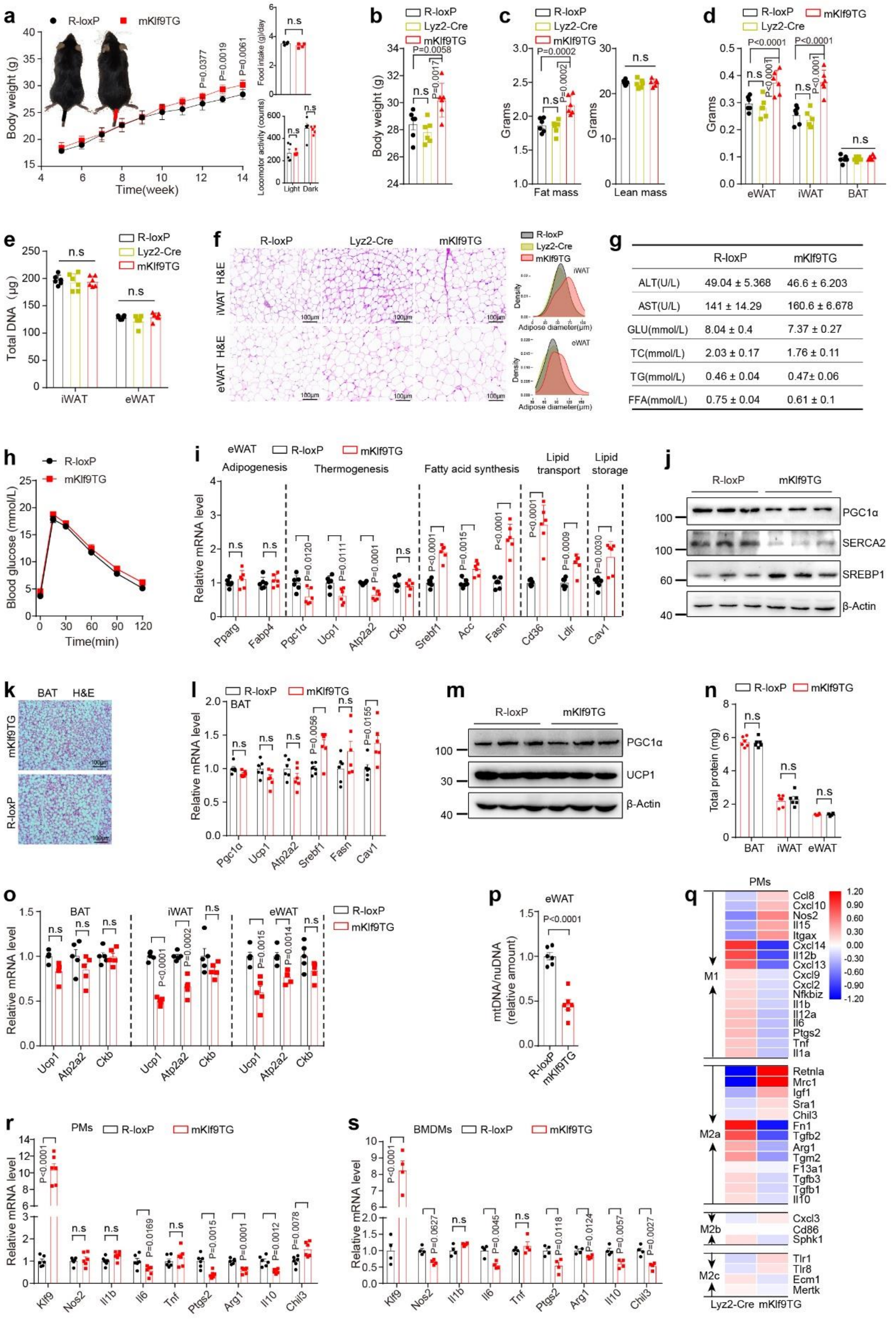

Fig. S4: Myeloid *Klf9* transgene induces adiposity and macrophage deactivation.

a, Body weight, food intake, and locomotor activity of male R-loxP and mKlf9TG mice fed an NCD for 14 weeks (n = 6 mice).

b, Body weight of 14-week-old male R-loxP, Lyz2-Cre, and mKlf9TG mice fed an NCD.

c-e, Fat and lean mass (**c**), AT weight (**d**), and total DNA content in iWAT and eWAT (**e**) of 14-week-old male R-loxP, Lyz2-Cre, and mKlf9TG mice (n = 6 mice).

f, H&E staining of iWAT and eWAT from male R-loxP, Lyz2-Cre, and mKlf9TG mice, scale bar = 100 μm, quantification of adipocyte size of iWAT and eWAT are also shown. Representative images of three independent experiments with similar results.

g, Serum ALT, AST, GLU, TG, TC, and FFA contents of male R-loxP and mKlf9TG mice (n = 5 mice).

h, GTT results of male R-loxP (n = 6 mice) and mKlf9TG (n = 5 mice) mice.

i, **j**, mRNA (n = 6 mice) (**i**) and protein (**j**) levels of indicated genes in eWAT of male R-loxP and mKlf9TG mice.

k, Representative H&E staining of BAT from male R-loxP and mKlf9TG mice, scale bar = 100 μm. Representative images of three independent experiments with similar results.

l, **m**, mRNA (n = 6 mice) (**l**) and protein (**m**) levels of indicated genes in BAT of male R-loxP and mKlf9TG mice.

n, Quantification of total protein per depot of indicated AT from male R-loxP and mKlf9TG mice (n = 6 mice).

o, mRNA levels indicated genes in indicated AT of male R-loxP and mKlf9TG mice during cold exposure (4°C) for 48h (n = 5 mice).

p, mtDNA copy number in eWAT of male R-loxP and mKlf9TG mice (n = 6 mice).

q, Heat map of indicated genes in PMs of male Lyz2-Cre and mKlf9TG mice.

r, **s**, mRNA levels of indicated genes in PMs (**r**) and BMDMs (**s**) from male R-loxP and mKlf9TG mice (n = 4 mice).

Data are represented as mean ± SEM., unpaired two-tailed Student's t tests were performed in **a**, **g**, **h**, **i**, **l**, **n**, **o**, **p**, **r**, and **s**, or one-way ANOVAs were performed in **b-e**.

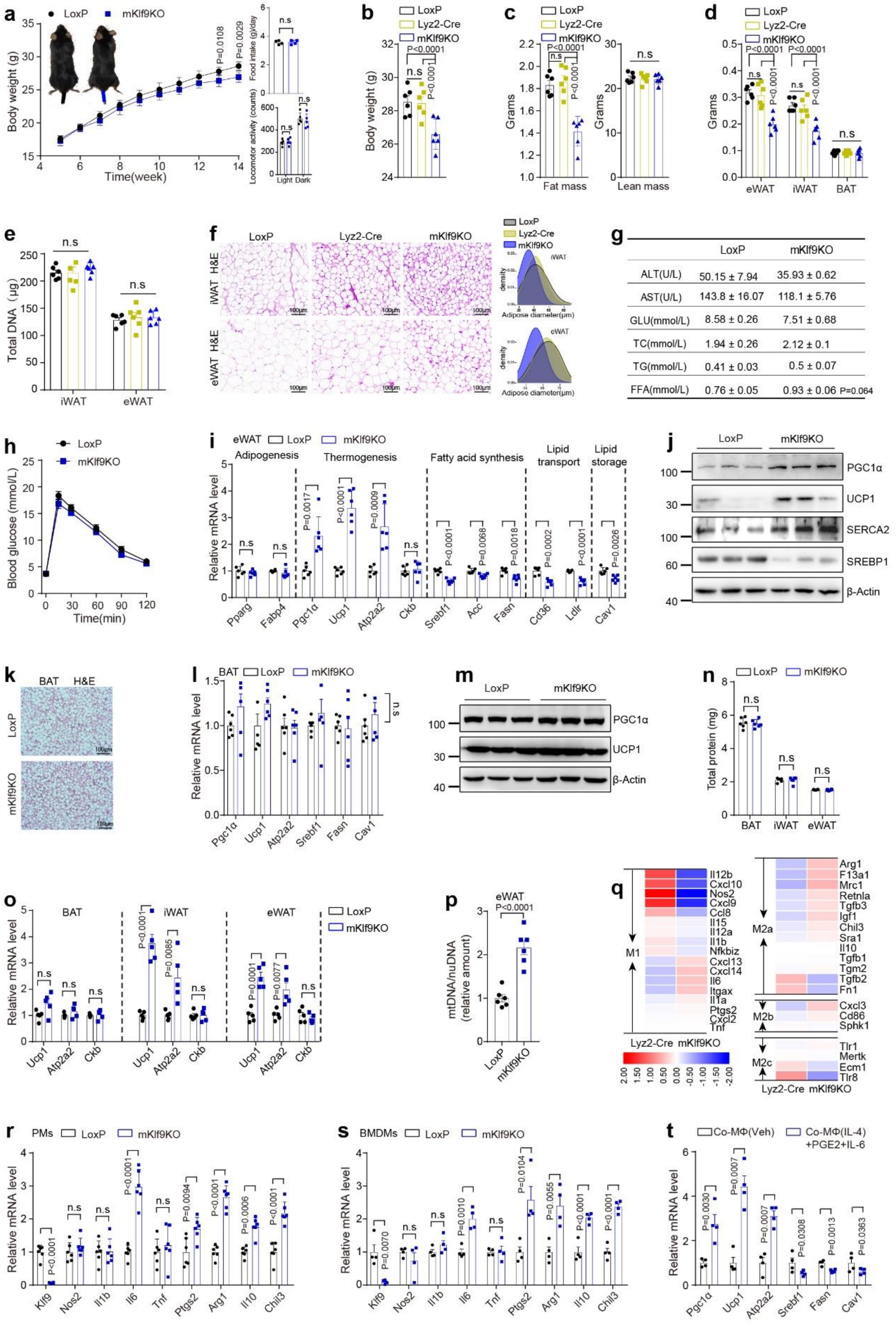

Fig. S5: Myeloid *Klf9* abrogation attenuates obesity, stimulates M2a macrophage activation, and increases the production of IL-6 and PGE2.

a, Body weight, food intake, and locomotor activity of male LoxP and mKlf9KO mice fed an NCD for 14 weeks (n = 6 mice).

b, Body weight of 14-week-old male LoxP, Lyz2-Cre, and mKlf9KO mice fed an NCD.

c-**e**, Fat and lean mass (**c**), AT weight (**d**) , and total DNA content in iWAT and eWAT (**e**) of 14-week-old male LoxP, Lyz2-Cre, and mKlf9KO mice (n = 6 mice).

f, H&E staining of iWAT and eWAT from male LoxP, Lyz2-Cre, and mKlf9KO mice, scale bar = 100 μm, quantification of adipocyte size are also shown. Representative images of three independent experiments with similar results.

g, Serum ALT, AST, GLU, TG, TC, and FFA contents of male LoxP and mKlf9KO mice (n = 5 mice).

h, GTT result of male LoxP (n = 5 mice) and mKlf9KO (n = 6 mice) mice.

i, **j**, mRNA (n = 6 mice) (**i**) and protein (**j**) levels of indicated genes in eWAT of male LoxP and mKlf9KO mice.

k, Representative H&E staining of BAT, scale bar = 100 μm. Representative images of three independent experiments with similar results.

l, **m**, mRNA (n = 6 mice) (**l**) and protein (**m**) levels of indicated genes in BAT of male LoxP and mKlf9KO mice.

n, Total protein per depot of indicated AT of male LoxP and mKlf9KO mice (n = 6 mice).

o, mRNA levels of indicated genes in indicated AT of male LoxP and mKlf9KO mice during cold exposure (4°C) for 48h (n = 5 mice).

p, mtDNA copy number in eWAT of male LoxP and mKlf9KO mice (n = 6 mice).

q, Heat map of indicated genes in PMs of male Lyz2-Cre and mKlf9KO mice.

r, **s**, mRNA levels of indicated genes in PMs (**r**) and BMDMs (**s**) of male LoxP and mKlf9KO mice (n = 4 mice).

t, mRNA levels of the indicated genes in beige adipocytes treated with CM containing IL-6 (0.2 ng/ml) and PGE2 (1μM) from indicated macrophages stimulated with IL-4 (20 ng/ml) (n = 4 independent experiments).

Data are represented as mean ± SEM., unpaired two-tailed Student's t tests were performed in **a**, **g**, **h**, **i**, **l**, **n**, **o**, **p**, and **r**-**t**, or one-way ANOVAs were performed in **b**-**e**.

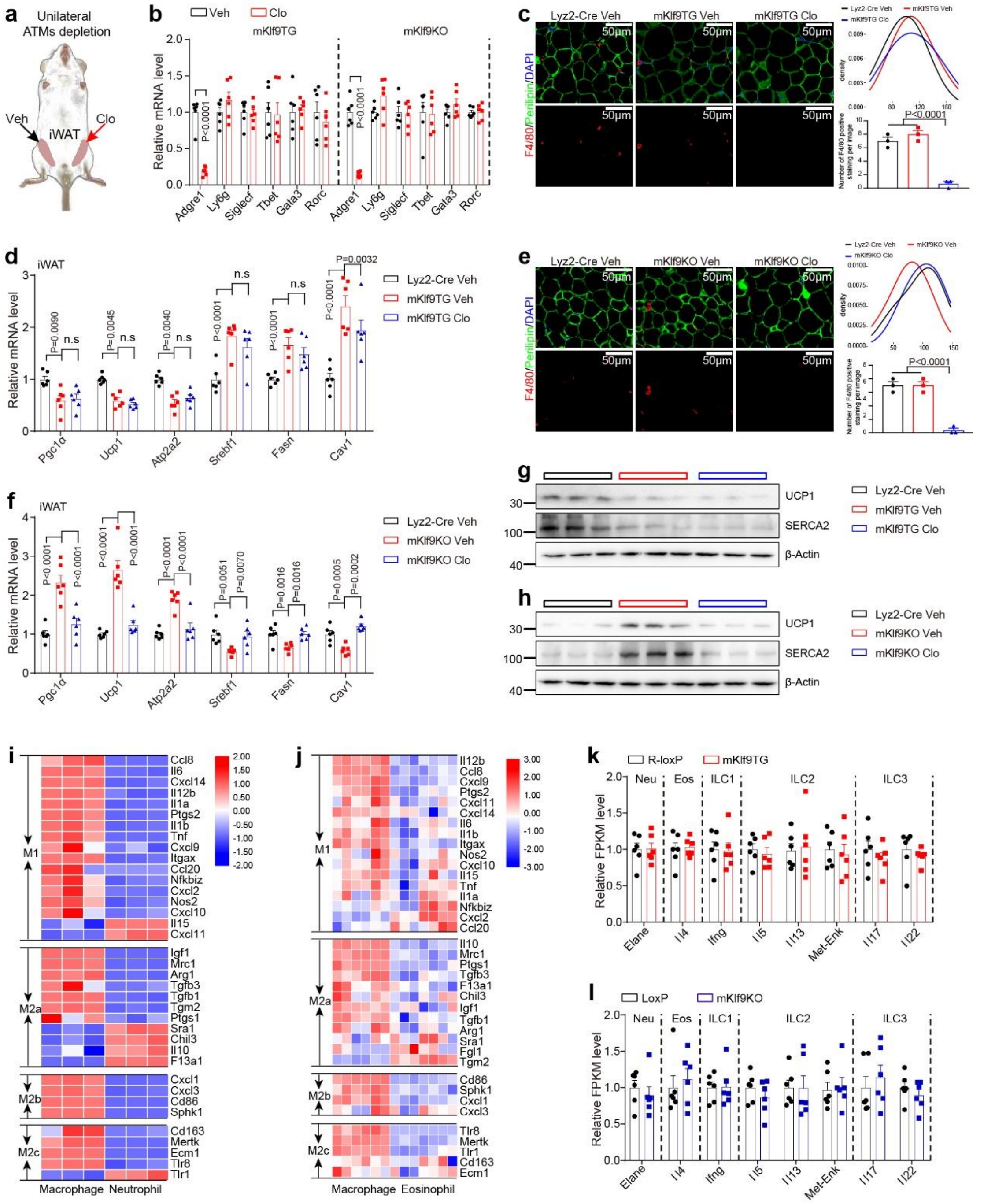

Fig. S6: Macrophage KLF9 regulates white adipose tissue energy homeostasis.

a, iWAT of 8-12 week-old mice was unilaterally delivered clodronate liposomes or control liposomes (Veh).

b, *Adgre1* (macrophages), *Ly6g* (neutrophils), *Siglecf* (eosinophils), *Tbet* (ILC1), *Gata3* (ILC2) and *Rorc* (ILC3) mRNA levels in iWAT of male mKlf9TG and mKlf9KO mice treated as in (**a**) (n = 6 mice).

c, Immunofluorescence staining of Perilipin-1 in iWAT of male Lyz2-Cre and mKlf9TG mice treated as in (**a**), scale bar = 50 μm, quantification of iWAT adipocyte size, and F4/80 positive staining per image are also shown (n = 3 mice). Representative images of three independent experiments with similar results.

d, **g**, mRNA (n = 6 mice) (**d**) and protein (**g**) levels of indicated genes in iWAT of male Lyz2-Cre and mKlf9TG mice treated as in (**a**).

e, Immunofluorescence staining of Perilipin-1 in iWAT of male Lyz2-Cre and mKlf9KO mice treated as in (**a**), scale bar = 50 μm, quantification of iWAT adipocyte size, and F4/80 positive staining per image are also shown (n = 3 mice). Representative images of three independent experiments with similar results.

f, **h**, mRNA (n = 6 mice) (**f**) and protein (**h**) levels of indicated genes in iWAT of male Lyz2-Cre and mKlf9KO mice treated as in (**a**).

i, Heat map representation of macrophage polarization markers in macrophages and neutrophils, data from GSE93735.

j, Heat map representation of macrophage polarization markers in macrophages and eosinophils, data from GSE112922.

k, mRNA levels of the indicated genes of iWAT from male R-loxP and mKlf9TG mice (n = 6 mice).

l, mRNA levels of the indicated genes of iWAT from male LoxP and mKlf9KO mice (n = 6 mice).

Data are represented as mean ± SEM., unpaired two-tailed Student's t tests were performed in **b**, **k**, and **l**, one-way ANOVAs were performed in **c** and **e**, or two-way ANOVAs were performed in **d** and **f**.

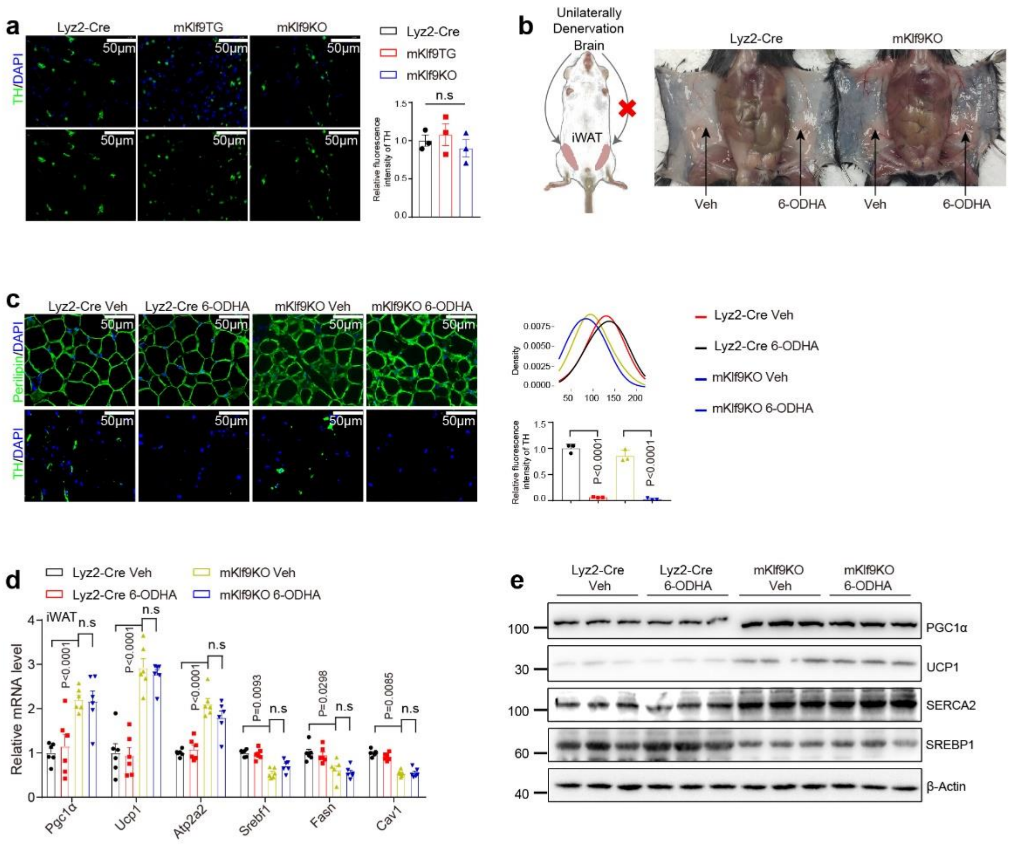

Fig. S7: Macrophage KLF9 regulates adipose tissue energy homeostasis independently of the nervous system.

a, Immunofluorescence staining of tyrosine hydroxylase (TH) in iWAT of male Lyz2-Cre, mKlf9TG, and mKlf9KO mice, scale bar = 50 μm, relative TH immunofluorescence intensity is also shown (n = 3 mice). Representative images of three independent experiments with similar results.

b, iWAT of 8-12 week old mice was unilaterally denervated (6-ODHA).

c, Immunofluorescence staining of Perilipin-1 and TH in iWAT of male Lyz2-Cre and mKlf9KO mice treated as in (**b**), scale bar = 50 μm, quantification of iWAT adipocyte size, and relative TH immunofluorescence intensity are also shown (n = 3 mice). Representative images of three independent experiments with similar results.

d, **e**, mRNA (**d**) (n = 6 mice) and protein (**e**) levels of indicated genes in iWAT of male Lyz2-Cre and mKlf9KO mice treated as in (**b**).

Data are represented as mean ± SEM., one-way ANOVAs were performed in **a** and **c**, or two-way ANOVAs were performed in **d**.

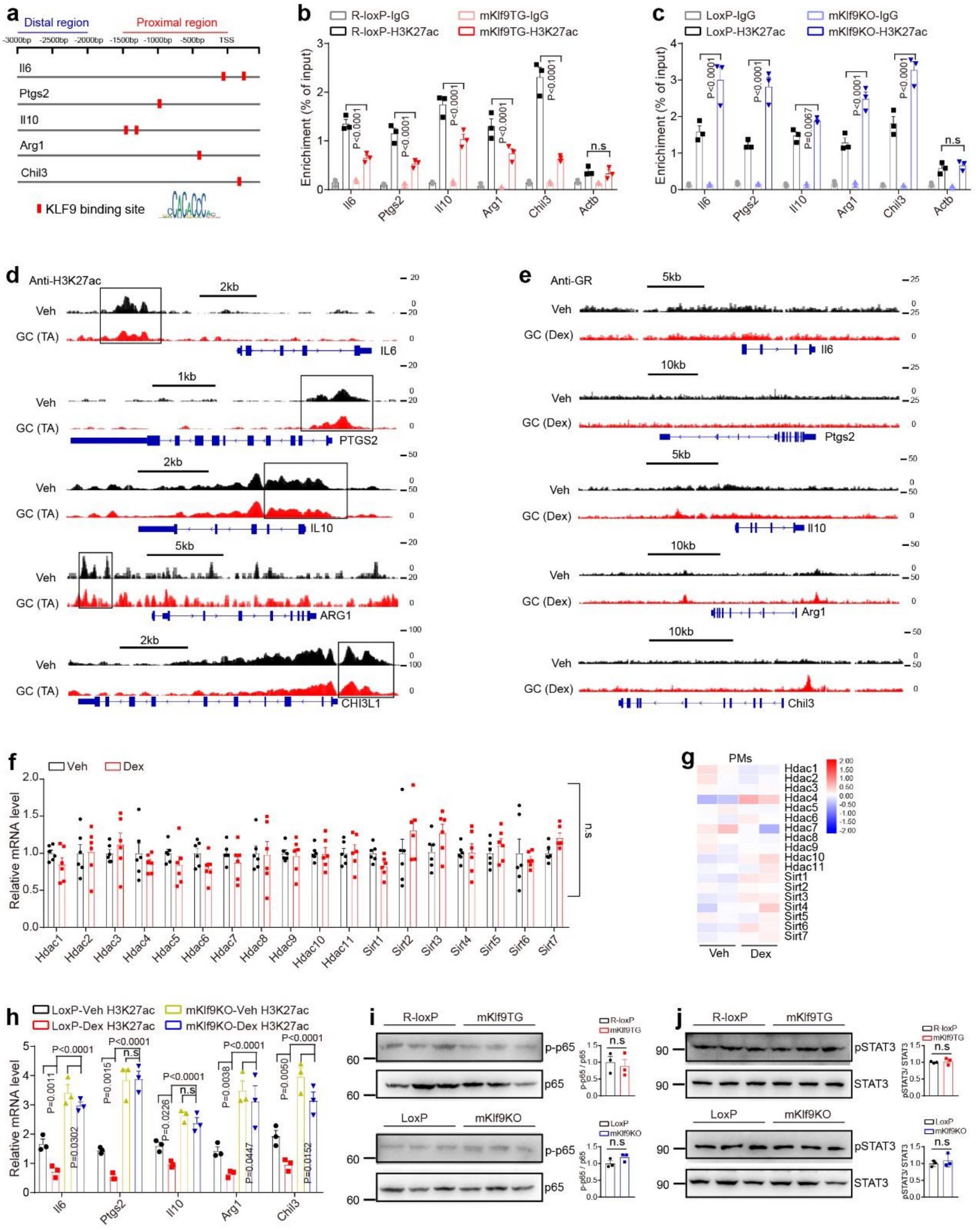

Fig. S8: Glucocorticoids reduce histone acetylation levels in the promoter regions of the M1 and M2a macrophage-associated marker genes.

a, KLF9 binding site analysis of indicated genes.

b, **c**, ChIP assay analysis of histone H3K27 acetylation levels in the promoters of indicated genes in mPMs from indicated mice co-stimulated with IL-4 (20 ng/ml) and LPS (1 ng/ml) for 12 h (n = 3 independent experiments).

d, Genome browser shots of H3K27ac ChIP-seq on indicated genes loci in MDMs treated with TA (1 μM) or vehicle. Genomic coordinates in hg19, data from GSE109440.

e, Genome browser shots of GR ChIP-seq on indicated genes loci in mPMs treated with Dex (100 nM) or vehicle. Genomic coordinates in mm10, data from GSE109131.

f, mRNA levels of HDACs in mPMs treated with Dex or vehicle (n = 6 mice).

g, Heat map representation of HDACs expression in mPMs treated with Dex or vehicle, data from GSE93735.

h, ChIP assay showing the H3K27ac levels in the promoters of indicated genes in mPMs from indicated mice treated with Dex (100 nM) for 4 h and then co-stimulated with IL-4 (20 ng/ml) and LPS (1 ng/ml) for 12 h (n = 3 independent experiments).

i, **j**, p65 and STAT3 phosphorylation levels in the indicated mPMs.

Data are represented as mean ± SEM., unpaired two-tailed Student's t tests were performed in **f**, **i**, and **j**, or one-way ANOVAs were performed in **b**, **c**, and **h**.

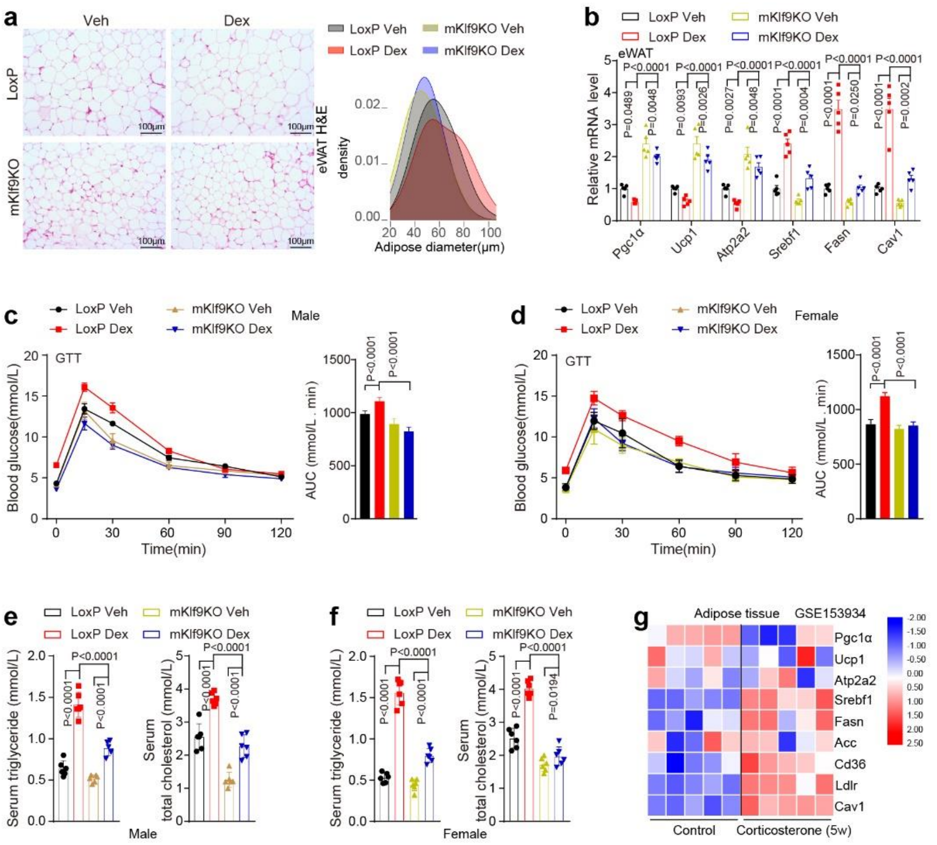

Fig. S9: Myeloid *Klf9* ablation prevents Dex-induced metabolic disorder.

a, Representative images of H&E staining of eWAT of male LoxP and mKlf9KO mice treated with vehicle or Dex (5 mg/kg) for 6 weeks (left), scale bar = 100 μm, quantification of adipocyte size of eWAT is also shown (right). Representative images of three independent experiments with similar results.

b, mRNA levels of indicated genes in eWAT of male LoxP and mKlf9KO mice treated as in (**a**) (n = 4 mice).

c, **d**, GTT result of male (**c**) and female (**d**) LoxP and mKlf9KO mice treated as in (**a**), quantification of area under the curve (AUC) is also shown (n = 6 mice).

e, **f**, Serum TG (left) and TC (right) contents in male (**e**), female (**f**) LoxP and mKlf9KO mice treated as in (**a**) (n = 6 mice).

g, Heat map representation of indicated genes in visceral adipose tissue treated with Cort or vehicle, data from GSE153934.

Data are represented as mean ± SEM., one-way ANOVAs were performed in **c**, **d**, **e** and **f**, or two-way ANOVAs were performed in **b**.

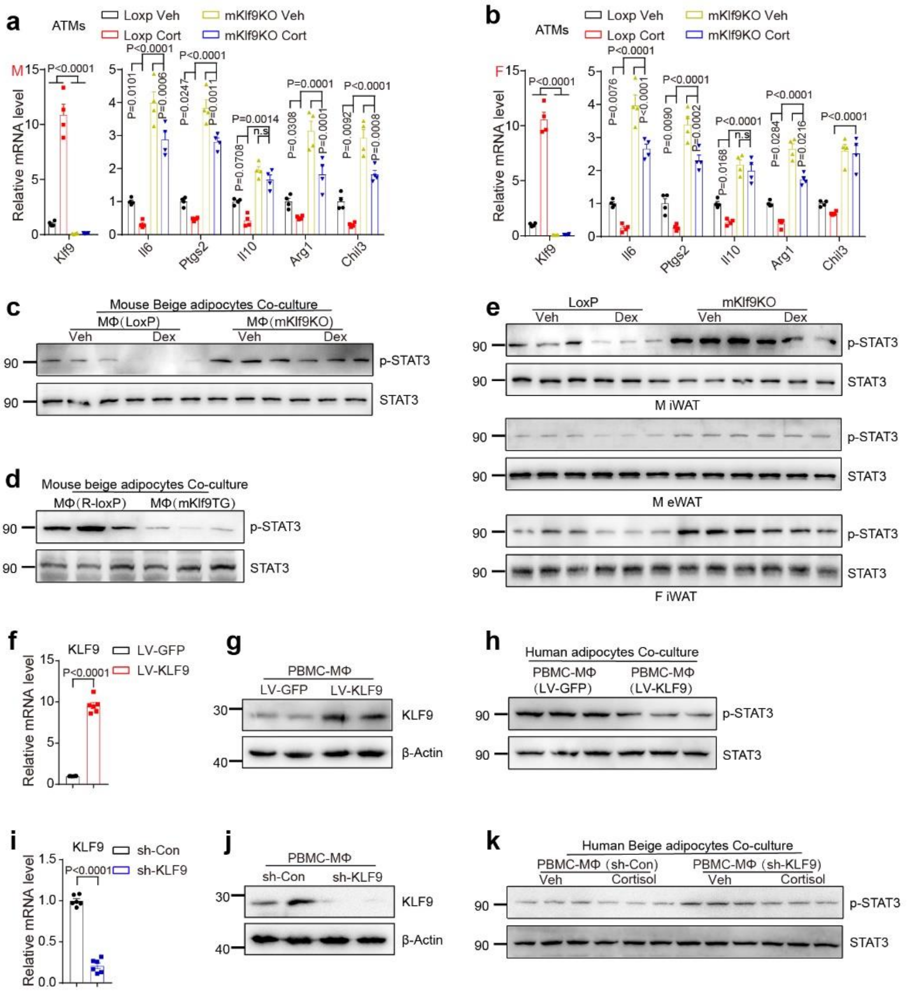

Fig. S10: Macrophage KLF9 controls beige adipocyte STAT3 signaling pathway.

a, **b**, mRNA levels of indicated genes in ATMs of male (**a**) and female (**b**) LoxP and mKlf9KO mice treated with 50 μg/mL Cort or corresponding vehicle (0.25% EtOH) in their drinking water for 6 weeks (n = 4 mice).

c, **d**, STAT3 phosphorylation in mouse beige adipocytes treated with CM from indicated mPMs.

e, STAT3 phosphorylation in iWAT and eWAT from male and female LoxP and mKlf9KO mice treated with Dex (5 mg/kg) or vehicle control for 6 weeks.

f, **g**, mRNA(n = 6 independent experiments) (**f**) and protein (**g**) levels of KLF9 in hPBMC-MΦ infected with LV-GFP or LV-KLF9 for 36 h.

h, STAT3 phosphorylation in human beige adipocytes treated with CM from hPBMC-MΦ pretreated with LV-KLF9 or LV-GFP for 24 h and then co-stimulated with IL-4 (20 ng/ml) and LPS (1 ng/ml) for another 12 h.

i, **j**, mRNA (n = 6 independent experiments) (**i**) and protein (**j**) levels of KLF9 in hPBMC-MΦ infected with sh-Con or sh-KLF9 for 36 h.

k, STAT3 phosphorylation in human beige adipocytes treated with CM from hPBMC-MΦ pretreated with sh-Con or sh-KLF9 for 24 h and then treated with cortisol (1μM) or vehicle for another 4 h and then co-stimulated with IL-4 (20 ng/ml) and LPS (1 ng/ml) for another 12 h.

Data are represented as mean ± SEM., unpaired two-tailed Student's t tests were performed in **f** and **i**, one-way ANOVAs were performed in **a** (left) and **b** (left), or two-way ANOVAs were performed in **a** (right) and **b** (right).

Supplementary Table 1

List of specific primers used for quantitative PCR gene expression analysis and ChIP analysis

Primers for quantitative PCR analysis

Gene symbol	Forward primer (5'→ 3')	Reverse primer (5'→ 3')
mKlf9	GCACAAGTGCCCCTACAGT	TGTATGCACTCTGTAATGGGCTTT
mPpargc1a	TGATGTGAATGACTTGGATACAGACA	GCTCATTGTTGTACTGGTTGGATATG
mUcp1	AGGCTTCCAGTACCATTAGGT	CTGAGTGAGGCAAAGCTGATTT
mAtp2a2	ACCTTTGCCGCTCATTTTCCAG	AGGCTGCACACACTCTTTACC
mCkb	ATCGAGAAGCTGGCAGTAGA	TGCTCCGCCTCAGTCATG
mSrebf1	GGAGCCATGGATTGCACATT	GGCCAGGGAAGTCACTGT
mAcc	AGGAAGATGGCGTCCGCTCTG	GGTGAGATGTGCTGGGTCAT
mFasn	AGGGGTCGACCTGGTCCTCA	GCCATGCCCAGAGGGTGGTT
mCd36	CCTCCAGAATCCAGACAACC	CACAGGCTTTCCTTCTTTGC
mVldlr	CTCCACCAACCTGCGGAGCC	GCTGACGGCCACACTGCTCA
mCav1	GACCCCAAGCATCTCAACGAC	AGACAACAAGCGGTAAAACCA
mNos2	CCTCATGCCATTGAGTTCATC	CCTGTTGTTTCTATTTCCTTTGTT
mIl1b	TGAAGTTGACGGACCCCAAAA	TGATGTGCTGCTGCGAGATT
mIl6	AGTTGCCTTCTTGGGACTGA	TCCACGATTTCCCAGAGAAC
mTnf	CGTCAGCCGATTTGCTATCT	CGGACTCCGCAAAGTCTAAG
mPtgs2	GAGCACAGGATTTGACCAGTAT	GGCTTCAGCAGTAATTTGATTC

mIl10	TGGACAACATACTGCTAACCGAC	CCTGGGGCATCACTTCTACC
mArg1	GCATATCTGCCAAAGACATCG	TCTTCCATCACCTTGCCAATC
mChil3	CATTGGAGGATGGAAGTTTGGA	GACGAAGGAATCTGATAACTGA
mElane	ACTTCGTCATGTCAGCAGCCCACT	GCTCCTGTCGCCGCAGGTCA
mIl4	AGACTCTTTCGGGCTTTTCG	TGATGCTCTTTAGGCTTTCC
mIfng	CAAGTGGCATAGATGTGGAA	TAATCTGGCTCTGCAGGATT
mIl5	ATGAGCACAGTGGTGAAAGA	TATGAGTAGGGACAGGAAGC
mIl13	GTGCAACGGCAGCATGGTAT	GGAGATGTTGGTCAGGGAAT
mIl33	GGAAAGAACCCACGAAAAGA	CTAGAATCCCGTGGATAGGC
mPenk	CGCCCAGGCGACATCAATTT	TCCTTGCAGGTCTCCCAGAT
mIl17	AGACTACCTCAACCGTTCCA	GAGCTTCCCAGATCACAGAG
mIl22	AAACTGTTCCGAGGAGTCAG	AGAACGTCTTCCAGGGTGAA
mHdac1	GGGCACCAAGAGGAAAGTC	GCAAATTGTGAGTCATGCG
mHdac2	AACTTGCTGCTAAATTATGGTTT	ACTTGATATACTCATCGCTGTGG
mHdac3	CGTATTTCTACGACCCCGAT	GGCTATGAGTCAATGCCAGG
mHdac4	ACAAGGAGAAGGGCAAAGAG	GGAAATGCAGTGGTTCAGGT
mHdac5	AATCCTGCCACGGACTCCT	GCTACCTCCACCTCCACCC
mHdac6	ATTCCTGTTGTCCAAGTCAAA	ACTCTGGTCCAAAGAAGCGTG
mHdac7	CAATAAAGACAAGAGCAAGCG	GATGGACTGTTCTCTCAAGGG

mHdac8	CGGTTTATATTTACAGTCCCG	GCATAGGCTTCGATCAGAGAG
mHdac9	TCAAGATAGCAAGGATGATTTC	ACTTTCTGTTTTAACCTGGACC
mHdac10	AGGAAGAGTTGGGCTTGGTG	TGCTTAGACAGTGCGTGGAG
mHdac11	GGAAATGGGGCAAGGTGAT	CAGCAGGTCCTCCTCCGAG
mSirt1	GTATTTTAGAAAAGACCCAAGACC	GTATTTTAGAAAAGACCCAAGACC
mSirt2	GATTCAGACTCGGACACTGAGG	TCGTCTAGAAGACGCTCCTTTT
mSirt3	GTAGGGTGGTGGTCATGGTG	CTGAAGGTTGCTGTAGAGGC
mSirt4	CGTGGACGCTTTGCACTCC	TGCTCCCCACAGTTCAGGC
mSirt5	GCCTCCCCACAAAGCAAGAT	TGGCGTTCGCAAAACACTTC
mSirt6	ACCATTCTGGACTGGGAGGA	GGTGACAGACAGGTCTGCGG
mSirt7	AGGCACTTGGTTGTCTACACG	CATACTCCATTAGGACCCCGA
m36B4	GAGGAATCAGATGAGGATATGGGA	AAGCAGGCTGACTTGGTTGC
hNOS2	AGAGGACCCAGGGACAAGCC	TTGTTTCTATCTCCTTTGTTACCG
hIL1B	TGACCTGAGCACCTTCTTTCC	GCACATAAGCCTCGTTATCCC
hIL6	GGCAGAAAACAACCTGAACCT	AACTCCAAAAGACCAGTGATG
hTNF	CACGCTCTTCTGCCTGCTG	GGCTTGTCACTCGGGGTTC
hPTGS2	AACTCTGGCTAGACAGCGTAA	AACCGTAGATGCTCAGGGAC
hARG1	AAGGGACAGCCACGAGGAG	TGTCAGCAAAGGGCAGGTC
hIL10	TGTTAAAGGAGTCCTTGCTGG	CTTGATGTCTGGGTCTTGGTT
hKLF9	TCCTCCCATCTCAAAGCCCATTA	ACAGCGGACAGCGGAACTGC

hPPARGC1A	GAACAAGACTATTGAACGCACCT	CTTGGTTGGCTTTATGAGGAG
hUCP1	CTCAGGATCGGCCTCTACG	CTTTCACGACCTCTGTGGG
hATP2A2	CTTGCTGGAACTTGTGATTG	CAAAGGCTGTAATTGTTTCTTC
hSREBF1	AGCTTCTCCATCAGTTCCAGC	TCAGAGAGGCCCACCACTTG
hFASN	GTCACCATCTCGGGACCTCA	GCACCTCCTTGGCAAACAC
hCAV1	GCGACCCTAAACACCTCAA	CTTCCAAATGCCGTCAAAA
h36B4	GTGTTCACCAAGGAGGACC	TGGCACAGTGACTTCACATG

Primers for ChIP analysis

mIl6 AceH3 (-622 to -531)	TTGGAGGTGAACAAACCATTAG	ACCCAACCTGGACAACAGACAG
mPtgs2 AceH3 (-487 to -304)	GAAAGACTTCAACCTAATTCCACCAG	GGGATCTAAGGTCCTAACTAAGGGA
mIl10 AceH3 (-187 to -73)	CATTCCGACCAGTTCTTTAGCG	CAGGCTCCTCCTCCCTCTTCTA
mArg1 AceH3 (-797 to -662)	ATTGCTCCGTTTCGATTCTTCT	GTGTGCCAAGTGCTATTCTAGTTAA
mChil3 AceH3 (-149 to -46)	TGACTGAACTGGTGATAAAAGGTG	AATGGGAAGTTTGGAAAAGGAA
mIl6 KLF9 (-218 to -55)	GTGCTCATGCTTCTTAGGGCTAG	AAATCTTTGTTGGAGGGTGGG
mPtgs2 KLF9 (-1094 to -1008)	ATTCACGCCAAGAACGTACAGT	TGTGAGGATGGAGTAGCGAAAA
mIl10 KLF9 (-1379 to -1195)	GAAAGTGAAAGGGATGGAGGC	GCTGGCAGATCAGGATCAAGG

mArg1 KLF9 (-545 to -419)	TGTCAGGGAAATAAATGATGC	CCAGGTTACACTGTCTAGGAAA
mChil3 KLF9 (+39 to +179)	GAACTTCAGTCTTGCATGGTT	TTCCTGTTATGGCTGTGGTAC
mIl6 KLF9 (-2667 to -2864)	CTCCCATAATCAAATGCCAATC	TACAACATAACGCTATCCTTC
mPtgs2 KLF9 (-2080 to -2038)	CATCTTGATTTGGTTTGGGACA	AAATTCAGACCTGGAGGACAA
mIl10 KLF9 (-2554 to -2608)	TTGAATCCGCAGCTCCGACAG	GCTGGGTTTCAGGACAAGGGA
mArg1 KLF9 (-2559 to -2782)	TTGTGCCACTTTGGGTTGAG	GTTCTCCTTCAGGCGACCAT
mChil3 KLF9 (-2392 to -2555)	TTTTGACCAATTTCCTCCTT	GATGGCACTTATGCTGATGT
