# Supplementary material for: Glucocorticoids increase adiposity by stimulating Krüppel-like factor 9 expression in macrophages
Source: Nat Commun. 2024 Feb 8;15:1190. doi: 10.1038/s41467-024-45477-8 (PMC10853261; doi:10.1038/s41467-024-45477-8)
Supplement: Supplementary file 3 — Reporting Summary [file 41467_2024_45477_MOESM3_ESM.pdf]

nature portfolio

Corresponding author(s): Yongsheng Chang

Last updated by author(s): Jan 12, 2024

Reporting Summary

Nature Portfolio wishes to improve the reproducibility of the work that we publish. This form provides structure for consistency and transparency in reporting. For further information on Nature Portfolio policies, see our Editorial Policies and the Editorial Policy Checklist.

Statistics

For all statistical analyses, confirm that the following items are present in the figure legend, table legend, main text, or Methods section.

n/a	Confirmed	
☐	☒	The exact sample size (*n*) for each experimental group/condition, given as a discrete number and unit of measurement
☐	☒	A statement on whether measurements were taken from distinct samples or whether the same sample was measured repeatedly
☐	☒	The statistical test(s) used AND whether they are one- or two-sided<br>*Only common tests should be described solely by name; describe more complex techniques in the Methods section.*
☐	☒	A description of all covariates tested
☒	☐	A description of any assumptions or corrections, such as tests of normality and adjustment for multiple comparisons
☐	☒	A full description of the statistical parameters including central tendency (e.g. means) or other basic estimates (e.g. regression coefficient) AND variation (e.g. standard deviation) or associated estimates of uncertainty (e.g. confidence intervals)
☐	☒	For null hypothesis testing, the test statistic (e.g. *F*, *t*, *r*) with confidence intervals, effect sizes, degrees of freedom and *P* value noted<br>*Give P values as exact values whenever suitable.*
☒	☐	For Bayesian analysis, information on the choice of priors and Markov chain Monte Carlo settings
☒	☐	For hierarchical and complex designs, identification of the appropriate level for tests and full reporting of outcomes
☒	☐	Estimates of effect sizes (e.g. Cohen's *d*, Pearson's *r*), indicating how they were calculated

Our web collection on statistics for biologists contains articles on many of the points above.

Software and code

Policy information about availability of computer code

Data collection	1. quantitative PCR: Roche LightCycler® 96<br>2. Western bolt: Tanon 5200<br>3. MRI: Echomri.Combo-700<br>4. Metabolic cages: Columbus Instruments<br>5. Glucose tolerance test: One Touch Ultra; LifeScan Inc.<br>6. Immunofluorescence: Leica or Olympus confocal laser scanning microscope<br>7. Flow cytometry: BD FACSAria llu flow cytometer
Data analysis	1. Adipocyte Size: ImageJ (Version 1.53)<br>2. Western Blot: ImageJ (Version 1.53)<br>3. Immunofluorescence: ImageJ (Version 1.53)<br>4. Statistical analysis : Microsoft Excel (Version 2019), GraphPad Prism (Version 7.04), and IBM SPSS statistics (Version 21)<br>5. RNAseq heatmap and Venn diagram were performed by R (Version 4.2.2)<br>6. ChIPseq were performed by IGV (Version 2.16)

For manuscripts utilizing custom algorithms or software that are central to the research but not yet described in published literature, software must be made available to editors and reviewers. We strongly encourage code deposition in a community repository (e.g. GitHub). See the Nature Portfolio guidelines for submitting code & software for further information.

Data

Policy information about availability of data

All manuscripts must include a data availability statement. This statement should provide the following information, where applicable:

- Accession codes, unique identifiers, or web links for publicly available datasets
- A description of any restrictions on data availability
- For clinical datasets or third party data, please ensure that the statement adheres to our policy

The RNA-seq data used in this study are publicly available in the NCBI Gene Expression Omnibus repository under the accession numbers GSE37660, GSE133127, GSE93735, GSE119789, GSE47538, GSE135130, GSE93735, GSE112922, GSE225833, GSE8616, and GSE153934. The data used to generate the main results shown in the main figures and extended figures are available in source data. Source data are provided with this paper. All data supporting the findings of this study are available from the corresponding authors on reasonable request.

Research involving human participants, their data, or biological material

Policy information about studies with human participants or human data. See also policy information about sex, gender (identity/presentation), and sexual orientation and race, ethnicity and racism.

Reporting on sex and gender	Peripheral blood mononuclear cells (PBMCs) were isolated from healthy blood donors, aged over 18, sex including both male and female.
Reporting on race, ethnicity, or other socially relevant groupings	N/A
Population characteristics	N/A
Recruitment	N/A
Ethics oversight	The study was approved by the Ethics Committee of the second hospital of Shandong university.

Note that full information on the approval of the study protocol must also be provided in the manuscript.

Field-specific reporting

Please select the one below that is the best fit for your research. If you are not sure, read the appropriate sections before making your selection.

[x] Life sciences [ ] Behavioural & social sciences [ ] Ecological, evolutionary & environmental sciences

For a reference copy of the document with all sections, see nature.com/documents/nr-reporting-summary-flat.pdf

Life sciences study design

All studies must disclose on these points even when the disclosure is negative.

Sample size	Sample size was determined on experiment feasibility and material availability.
Data exclusions	No data was excluded.
Replication	All data at least three biological replicates with the n indicated in each experiment. All attempts at replication were successful.
Randomization	Cell samples and research animals were randomly assigned to treatment groups.
Blinding	The treatments provided to mice and cell were not blinded for practical reasons to avoid mix up. To prevent bias, the investigators were blinded to group allocation during data collection and analysis.

Reporting for specific materials, systems and methods

We require information from authors about some types of materials, experimental systems and methods used in many studies. Here, indicate whether each material, system or method listed is relevant to your study. If you are not sure if a list item applies to your research, read the appropriate section before selecting a response.

Materials & experimental systems

n/a	Involved in the study
☐	☒ Antibodies
☐	☒ Eukaryotic cell lines
☒	☐ Palaeontology and archaeology
☐	☒ Animals and other organisms
☒	☐ Clinical data
☒	☐ Dual use research of concern
☒	☐ Plants

Methods

n/a	Involved in the study
☒	☐ ChIP-seq
☐	☒ Flow cytometry
☒	☐ MRI-based neuroimaging

Antibodies

Antibodies used

KLF9 (Invitrogen; Cat: 701888; Clone name: 5H16L7; Dilution: 1:1000), KLF9 (Abcam; Cat: ab227920; Dilution: 1:1000), PGC1α (Millipore; Cat: AB3242; Dilution: 1:1000), UCP1 (Abcam; Cat: ab10983; Dilution: 1:1000), SERCA2(ABclonal; Cat: A11692; Clone name: ARC0679; Dilution: 1:1000), β-Actin (ABclonal; Cat: AC026; Clone name: ARC5115-01; Dilution: 1:20000), Acetyl-Histone H3 antibody (ABclonal; Cat: A17917), COX2 (ABclonal; Cat: A3560; Clone name: ARC0800; Dilution: 1:1000), ARG1 (ABclonal; Cat: A4923; Clone name: ARC1164; Dilution: 1:1000), SREBP1 (ABclonal; Cat: A15586; Dilution: 1:1000), HDAC1 (ABclonal; Cat: A0238; Dilution: 1:1000), HDAC2 (ABclonal; Cat: A2084; Dilution: 1:1000), SIN3A (Proteintech; Cat: 14638-1-AP; Dilution: 1:1000), STAT3 (Santa Cruz Biotechnology; Cat: sc-8019; Dilution: 1:50), P-STAT3 (Santa Cruz Biotechnology; Cat: sc-8059; Dilution: 1:50), p65 (Cell Signaling Technology; Cat: #8242; Clone name: D14E12; Dilution: 1:1000), P-p65 (Cell Signaling Technology; Cat: #3033; Clone name: 93H1; Dilution: 1:1000), Acetyl-Histone H3K27 antibody (ABclonal; Cat: A7253), F4/80 (Proteintech; Cat: 28463-1-AP; Dilution: 1:200), Perilipin-1 (Cell Signaling Technology; Cat: #9349; Clone name: D1D8; Dilution: 1:200), Tyrosine Hydrolase (Santa Cruz Biotechnology; Cat: sc-25269; Dilution: 1:50), CD16/CD32 (BioLegend; Cat: 101302; Clone name: 93; Dilution: 1:100), PE-conjugated anti-mouse CD11b (BioLegend; Cat: 101207; Clone name: M1/70; Dilution: 1:100), PE-conjugated anti-TREM2 (R&D Systems, Cat: FAB17291P; Dilution: 1:100), PE-conjugated anti-mouse CD11c (BioLegend; Cat: 117308; Clone name: N418; Dilution: 1:100), PE-conjugated anti-mouse CD206 (BioLegend; Cat: 141706; Clone name: C068C2; Dilution: 1:100), APC-conjugated anti-mouse F4/80 (BioLegend; Cat: 123116; Clone name: BM8; Dilution: 1:100), FITC-conjugated anti-mouse KLF9 (Biorbyt; Cat: orb9122; Dilution: 1:100).
Secondary antibodies: HRP Goat Anti-Rabbit IgG (H+L) (ABclonal; Cat: AS014; Dilution: 1:5000), HRP Goat Anti-Mouse IgG (H+L) (ABclonal; Cat: AS003; Dilution: 1:5000)
All antibodies were used according to the manufacturer's instructions.

Validation

All antibodies used in our study have been validated and detailed information could be found on the manufacturers' websites as listed below:

KLF9 (Invitrogen; 701888)
https://www.thermofisher.cn/antibody/primary/query/AB_2633037

KLF9 (Abcam; ab227920)
https://www.abcam.cn/products/primary-antibodies/klf9-antibody-ab227920.html

PGC1α (Millipore; AB3242)
https://www.sigmaaldrich.cn/CN/zh/search/ab3242?
focus=products&page=1&perpage=30&sort=relevance&term=ab3242&type=product

UCP1 (Abcam; ab10983)
https://www.abcam.cn/products/primary-antibodies/ucp1-antibody-ab10983.html

SERCA2(ABclonal; A11692)
https://abclonal.com.cn/catalog/A16975

β-Actin (ABclonal; AC026)
https://abclonal.com.cn/catalog/AC026

Acetyl-Histone H3 antibody (ABclonal; A17917)
https://abclonal.com.cn/catalog/A17917

COX2 (ABclonal; A3560),
https://abclonal.com.cn/catalog/A3560

ARG1 (ABclonal; A4923),
https://abclonal.com.cn/catalog/A4923

SREBP1 (ABclonal; A15586),
https://abclonal.com.cn/catalog/A15586

HDAC1 (ABclonal; A0238),

https://abclonal.com.cn/catalog/A0238

HDAC2 (ABclonal; A2084),
https://abclonal.com.cn/catalog/A2084

SIN3A (Proteintech; 14638-1-AP)
https://www.ptgcn.com/products/SIN3A-Antibody-14638-1-AP.htm

STAT3 (Santa Cruz Biotechnology; sc-8019),
https://www.scbt.com/p/stat3-antibody-f-2?requestFrom=search

P-STAT3 (Santa Cruz Biotechnology; sc-8059),
https://www.scbt.com/p/p-stat3-antibody-b-7?requestFrom=search

p65 (Cell Signaling Technology; #8242),
https://www.cellsignal.cn/products/primary-antibodies/nf-kb-p65-d14e12-xp-rabbit-mab/8242?site-search-type=Products&N=4294956287&Ntt=%238242&fromPage=plp&_requestid=368221

P-p65 (Cell Signaling Technology; #3033),
https://www.cellsignal.cn/products/primary-antibodies/phospho-nf-kb-p65-ser536-93h1-rabbit-mab/3033?site-search-type=Products&N=4294956287&Ntt=%233033&fromPage=plp&_requestid=368398

Acetyl-Histone H3K27 antibody (ABclonal; A7253)
https://abclonal.com.cn/catalog/A7253

F4/80 (Proteintech; 28463-1-AP),
https://www.ptgcn.com/products/F4-80-Antibody-28463-1-AP.htm

Perilipin-1 (Cell Signaling Technology; #9349)
https://www.cellsignal.cn/products/primary-antibodies/perilipin-1-d1d8-xp-rabbit-mab/9349?site-search-type=Products&N=4294956287&Ntt=%239349&fromPage=plp&_requestid=352673

TH (Santa Cruz Biotechnology; sc-25269)
https://www.scbt.com/p/th-antibody-f-11?requestFrom=search

CD16/CD32 (BioLegend; 101302),
https://www.biolegend.com/en-us/products/purified-anti-mouse-cd16-32-antibody-190

PE-conjugated anti-mouse CD11b (BioLegend; 101207),
https://www.biolegend.com/en-us/products/pe-anti-mouse-human-cd11b-antibody-349

PE-conjugated anti-TREM2 (R&D Systems, FAB17291P)
https://www.rndsystems.com/cn/products/human-mouse-trem2-pe-conjugated-antibody-237920_fab17291p

PE-conjugated anti-mouse CD11c (BioLegend; 117308)
https://www.biolegend.com/en-us/products/pe-anti-mouse-cd11c-antibody-1816

PE-conjugated anti-mouse CD206 (BioLegend; 141706)
https://www.biolegend.com/en-us/products/pe-anti-mouse-cd206-mmr-antibody-7424

APC-conjugated anti-mouse F4/80 (BioLegend; 123116),
https://www.biolegend.com/en-us/products/apc-anti-mouse-f4-80-antibody-4071

FITC-conjugated anti- mouse KLF9 (Biorbyt; orb9122).
https://www.biorbyt.com/klf9-antibody-fitc-orb9122.html

HRP Goat Anti-Rabbit IgG (H+L) (ABclonal; AS014)
https://abclonal.com.cn/catalog/AS014

HRP Goat Anti-Mouse IgG (H+L) (ABclonal; AS003)
https://abclonal.com.cn/catalog/AS003

Eukaryotic cell lines

Policy information about cell lines and Sex and Gender in Research

Cell line source(s)	1. HEK293 cells were bought from ATCC (CRL-3216).

	2. THP-1 cells were bought from Pricella (CL-0233)<br>3. Human adipose-derived stem cells (ADSCs) were bought from Pricella (CP-H202)
Authentication	None of the cell lines were authenticated
Mycoplasma contamination	All cell lines were tested negative for mycoplasma contamination.
Commonly misidentified lines (See ICLAC register)	No commonly misidentified cell lines were used.

Animals and other research organisms

Policy information about studies involving animals; ARRIVE guidelines recommended for reporting animal research, and Sex and Gender in Research

Laboratory animals	Mice were maintained on the C57BL/6 background. Rosa26 Klf9 flox/flox mice were generated using the CRISPR/Cas9 system to insert the CAG-LoxP-STOP-LoxP-Klf9 cassette into the mouse Rosa26 locus. Klf9 flox/flox mice were also generated by CRISPR/Cas9 system. Lyz2-Cre mice was gift from Professor Hongbing Zhang (Institute of Basic Medical Sciences, Peking Union Medical College). Myeloid cell-specific Klf9 knock-in mice (mKlf9TG) were generated by breeding Lyz2-Cre mice with Rosa26 Klf9 flox/flox. Myeloid cell-specific Klf9 abrogation mice (mKlf9KO) were generated by breeding Lyz2-Cre mice with Klf9 flox/flox. All animals were housed at 21± 1 °C with a humidity of 50% ± 5% in a 12 h light/dark cycle and fed ad libitum with standard mouse feed and water throughout the experiments. Further, 8-14-week-old mice with different genotypes were used in our experiments. For cold exposure experiments, mice were placed in a refrigerator (4°C) with free access to food and water.
Wild animals	This study did not involve the use of wild animals
Reporting on sex	Only male mice were used in the mechanism study of myeloid KLF9 effect on adipose tissue biology. Both male and female mice were used to determine whether GC-inducible KLF9 in macrophages mediates the effects of GCs on adiposity.
Field-collected samples	The study did not involve samples collected from the field
Ethics oversight	The procedures related to animal subjects were approved by Institutional Animal Care and Use Committee of Tianjin Medical University.

Note that full information on the approval of the study protocol must also be provided in the manuscript.

Flow Cytometry

Plots

Confirm that:

- [x] The axis labels state the marker and fluorochrome used (e.g. CD4-FITC).
- [x] The axis scales are clearly visible. Include numbers along axes only for bottom left plot of group (a 'group' is an analysis of identical markers).
- [x] All plots are contour plots with outliers or pseudocolor plots.
- [x] A numerical value for number of cells or percentage (with statistics) is provided.

Methodology

Sample preparation	Cells were incubated with Fc Block (101302; BioLegend) and washed using PBS with 2% fetal calf serum, and then cells were stained with APC-conjugated anti-F4/80 (123116; BioLegend), PE-conjugated anti-CD11b (101207; BioLegend), PE-conjugated anti-mouse CD11c (BioLegend; 117308), PE-conjugated anti-mouse CD206 (BioLegend; 141706), PE-conjugated anti-TREM2 (R&D Systems, FAB17291P) or isotype antibodies. According to the manufacturer’s instructions, the intracellular staining of KLF9 (orb9122; Biorbyt) was performed with the transcription factor staining buffer set (eBioscience).
Instrument	Stained cells were analyzed with a BD FACSAria IIu flow cytometer
Software	The data was analyzed with FlowJo v10.7.0.
Cell population abundance	Target cell populations were determined by positive surface marker staining.
Gating strategy	Adipose tissue macrophages were identified as CD11b+F4/80+, M1 macrophages were identified as F4/80+CD11c+, M2 macrophages were identified as F4/80+CD206+, and LAM macrophages were identified as F4/80+TREM2+

- [ ] Tick this box to confirm that a figure exemplifying the gating strategy is provided in the Supplementary Information.
